# Drivers of ecological assembly in the hindgut of Atlantic Cod fed a macroalgal supplemented diet

**DOI:** 10.1038/s41522-022-00296-x

**Published:** 2022-05-04

**Authors:** C. Keating, M. Bolton-Warberg, J. Hinchcliffe, R. Davies, S. Whelan, A. H. L. Wan, R. D. Fitzgerald, S. J. Davies, C. J. Smith, U. Z. Ijaz

**Affiliations:** 1 Microbiology Discipline, School of Natural Sciences, National University of Ireland Galway, Galway, Ireland; 2 Water and Environment Group, Infrastructure and Environment Division, James Watt School of Engineering, University of Glasgow, Glasgow, UK; 3 Institute of Biodiversity, Animal Health & Comparative Medicine, University of Glasgow, Glasgow, UK; 4 Carna Research Station, Ryan Institute, National University of Ireland Galway, Carna Co. Galway, Ireland; 5 Department of Biological and Environmental Sciences, University of Gothenburg, Gothenburg, Sweden; 6 AquaBioTech Group, Central Complex, Targa Gap Mosta, Malta; 7 Irish Seaweed Research Group, Ryan Institute and School of Natural Sciences, National University of Ireland Galway, Galway, Ireland; 8 Aquaculture and Nutrition Research Unit, Carna Research Station, Ryan Institute and School of Natural Sciences, National University of Ireland Galway, Carna Co. Galway, Ireland; 9 Department of Animal Production, Welfare and Veterinary Science, Harper Adams University, Newport, Shropshire UK

**Keywords:** Applied microbiology, Microbiome, Microbial ecology

## Abstract

It is difficult to disentangle the many variables (e.g. internal or external cues and random events) that shape the microbiota in the gastrointestinal tract of any living species. Ecological assembly processes applied to microbial communities can elucidate these drivers. In our study, farmed Atlantic cod (*Gadus morhua*) were fed a diet of 10% macroalgae supplement (*Ulva rigida* [ULVA] or *Ascophyllum nodosum* [ASCO] or a non-supplemented control diet [CTRL]) over 12 weeks. We determined the influence of ecological assembly processes using a suite of null-modelling tools. We observed dissimilarity in the abundance of common OTUs over time, which was driven by deterministic assembly. The CTRL samples showed selection as a critical assembly process. While dispersal limitation was a driver of the gut microbiome for fish fed the macroalgae supplemented diet at Week 12 (i.e., ASCO and ULVA). Fish from the ASCO grouping diverged into ASCO_N (normal) and ASCO_LG (lower growth), where ASCO_LG individuals found the diet unpalatable. The recruitment of new taxa overtime was altered in the ASCO_LG fish, with the gut microbiome showing phylogenetic underdispersion (nepotistic species recruitment). Finally, the gut microbiome (CTRL and ULVA) showed increasing robustness to taxonomic disturbance over time and lower functional redundancy. This study advances our understanding of the ecological assembly and succession in the hindgut of juvenile Atlantic cod across dietary treatments. Understanding the processes driving ecological assembly in the gut microbiome, in fish research specifically, could allow us to manipulate the microbiome for improved health or resilience to disease for improved aquaculture welfare and production.

## Introduction

Aquaculture has become the fastest-growing food sector this century, surpassing over 82 million tonnes of seafood production in 2018^1^ contributing >45% of the global seafood production^1^. At present, however, Atlantic cod (*Gadus morhua*) is primarily harvested through capture fisheries owing to species-specific production bottlenecks in aquaculture, leading to reduced profitability^2,3^. Such bottlenecks include poor larval survival rates^4^, early sexual maturation (reduced fillet yield)^5^, and reduced fish growth^6^. A review of these challenges is noted here^7^. An avenue that is being increasingly explored is the addition of aquafeed ingredients from a variety of natural sources that provide additional health benefits to the growing fish (e.g., macroalgae supplements^8–10^, immunostimulants^11,12^, pre- and probiotics^13,14^). In conjunction with such diet supplements, it is apparent that the impact of these feeds on the gut microbiome and fish health should be considered.

The fish gut microbiome is increasingly used to elucidate fish condition^15^, response to environmental conditions^16^ and changing diet^17,18^ and has been extensively reviewed elsewhere^15,19,20^. It is a tantalising goal to use the information yielded by microbial ecology to manipulate a particular fish species’ gut microbiome for improved welfare and growth in a commercial setting. To be tenable, we need to understand the gut microbiome beyond a descriptive analysis of the species found within a specific condition. Taking an example from our previous work, over twelve weeks, we fed Atlantic cod juveniles (*G. morhua*) a diet of 10% macroalgal supplement either *Ascophyllum nodosum* (ASCO) or *Ulva rigida* (ULVA) species, or a control non-amended diet^21^. We showed that temporal pressures outweighed the response to diet supplementation, with the gut microbiome of all fish consuming the different diets converging. It is unclear if this shift resulted from natural community succession in the developing hindgut microbiome of the juvenile farmed fish or other factors. Indeed, little is known about the gut microbiome development in cod, with a limited number of studies that have only reported on the larval phase^22^ or wild adults^23^. Moreover, the microbial communities within the gut face various driving forces from the host, environmental factors, and individual species interactions, for example.

Ecological theory has been used to describe the mechanisms that shape community assembly. It is more typically applied to macroorganisms (e.g., fish species^24^) or a population of macroorganisms within a specific environment (e.g., shore communities^25^). This approach is increasingly being used in microbial ecology^26–28^ to determine underlying rules driving the assembly and dynamics of microbial communities, which is particularly challenging^29^. The gut microbiome is a complex and dynamic ecosystem subject to fluctuating abiotic and biotic conditions. Authors have noted that founder (priority) effects can play a key role in the community assembly and succession, i.e., the species that first colonise will alter the environment and determine the resulting community structure^30,31^. The colonisation of that first species may be random like a lottery^30^, or determined via resource availability and species traits^32,33^. Assembly processes are broadly described as either random (stochastic) or non-random (deterministic)^29^. Researchers have noted that four assembly processes can occur in microbial communities: neutral and stochastic processes (e.g., probabilistic events such as births, deaths, mutations and ecological drift^34^) or non-probable events driven from niche or deterministic forces (e.g., environmental conditions, species interactions, and traits). Neutral theory differs from niche-based theory in assuming that all species are equal in functional traits, demographic rates, and the environment does not select. A range of tools have been described to determine the underlying mechanisms of microbial community assembly. Many of these rely on ‘null models’. A null modelling approach considers randomising the original community structure and then, through a statistical framework, compares microbiome properties between the original and randomised communities to elucidate a particular ecological phenomenon. The randomised community is generated to mimic a community without the force of a specified assembly process^35^. Deviations from the null model can then be used to predict the processes occurring in the real community.

Microbial community assembly mechanisms themselves remain relatively underexplored within fish gut studies. A recent review by Derome and Filteau (2020) summarises the work that has been published so far and offers a perspective on future directions^36^. Exploring these mechanisms may provide a realistic opportunity to manipulate the gut microbiome to improve commercial fish conditions, increase disease resilience, and mitigate stress under aquaculture conditions. Therefore, in the present study, the aims are to implement a suite of ‘null-modelling’ tools to understand the microbial ecological assembly mechanisms in the gut of Atlantic cod (*G. morhua*) in an experimental feeding trial over twelve weeks. Specifically, we aim to determine: (i) whether the microbial community was driven by stochastic, deterministic forces, niche, or neutral effects, (ii) the influence of specific ecological processes on microbial diversity, (iii) colonisation and community succession mechanisms and finally, (iv) the resilience of the gut microbial function to taxonomic perturbation. We hypothesised that the gut microbiome would demonstrate deterministic and niche-based assembly linked to host development, that this would outweigh environmental variables such as feed type, and that individuals with poorer growth (ASCO_LG) from a subset of fish that found the diet unpalatable, would demonstrate vulnerabilities to taxonomic perturbation. Our work has significant importance with respect to fish gut microbiome research. More broadly, this work is relevant to areas where manipulating the microbial community through novel feed additives and functional supplements is desired.

## Results

**Fig. 1:**
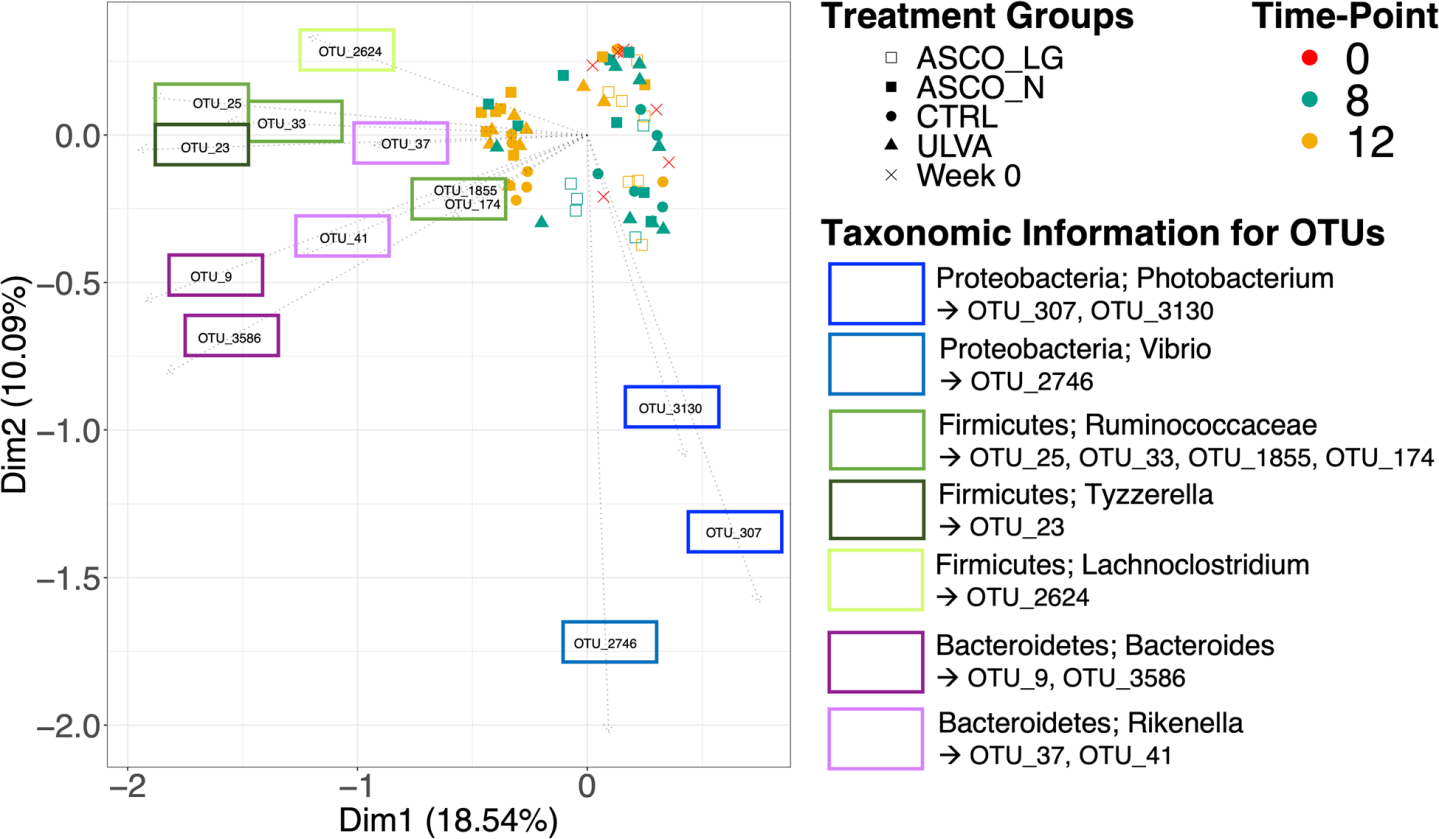
Temporal dissimilarity of the gut microbiota of Atlantic cod. Dissimilarity is explained by *Proteobacteria* species at Week 0/Week 8 and *Bacteroidetes* and *Firmicutes* species at Week 12. The points represent grouped samples with ‘Time’ denoted by colour; 0 (red), 8 (green), 12 (orange), and dietary treatments denoted by symbols. Deviance in ordination space is explained by 24.5%. PERMANOVA (sources of variability explained by dissimilarities between samples) showed that variation accounted for by Treatment Groups; R2 = 0.098, ***p = 0.001 and variation accounted for by Time-Point; R2 = 0.08644, ***p = 0.001.

It was evident that there were strong temporal pressures on the hindgut microbial community of juvenile Atlantic cod (*G. morhua*), with a change in the microbial community composition over the 12-week trial. Dissimilarity in community composition was highly significant across time points (Beta-diversity Bray-Curtis dissimilarity matrix - *p* = 0.001). The OTUs that best correlated with the temporal community dissimilarities were *Proteobacteria*, *Bacteroidetes* and *Firmicutes* species. *Proteobacteria* spp. (OTU_3130 + OTU_307 - *Photobacterium* spp. and OTU_2746 - *Vibrio* sp.) which were associated with Week 0 to Week 8 (Fig. 1). While *Bacteroidetes* spp. (OTU_9 + OTU_3586 - *Bacteroides* spp. and OTU_41 + OTU_37 - *Rikenella* spp.) and *Firmicutes* spp. (OTU_1855 + OTU_174 + OTU_33 - *Ruminococcaceae* spp., OTU_2624 - *Lachnoclostridium* sp. and OTU_23 - *Tyzzerella* sp.) correlated with Week 12 samples (Fig. 1). The OTU subset that most explained the dissimilarity pattern included all these OTUs except OTU_37 – *Rikenella* sp. (R = 0.902). Notably, a temporal shift in OTUs was not observed in the ASCO_LG individuals.

Predictive functional analysis using PICRUSt2 revealed that some of the detected microbial metabolism pathways had significantly changed (greater than Log_2_ fold) from Week 0 to Week 12 (Supplementary Fig. 1). This included pathways related to lysine biosynthesis and degradation and methane metabolism detected at Week 12, which were not found at Week 0.

### What microbial community assembly mechanisms are shaping the microbiome of juvenile Atlantic cod (*Gadus morhua*), and how are these influenced by macroalgal feed supplements?

**Fig. 2:**
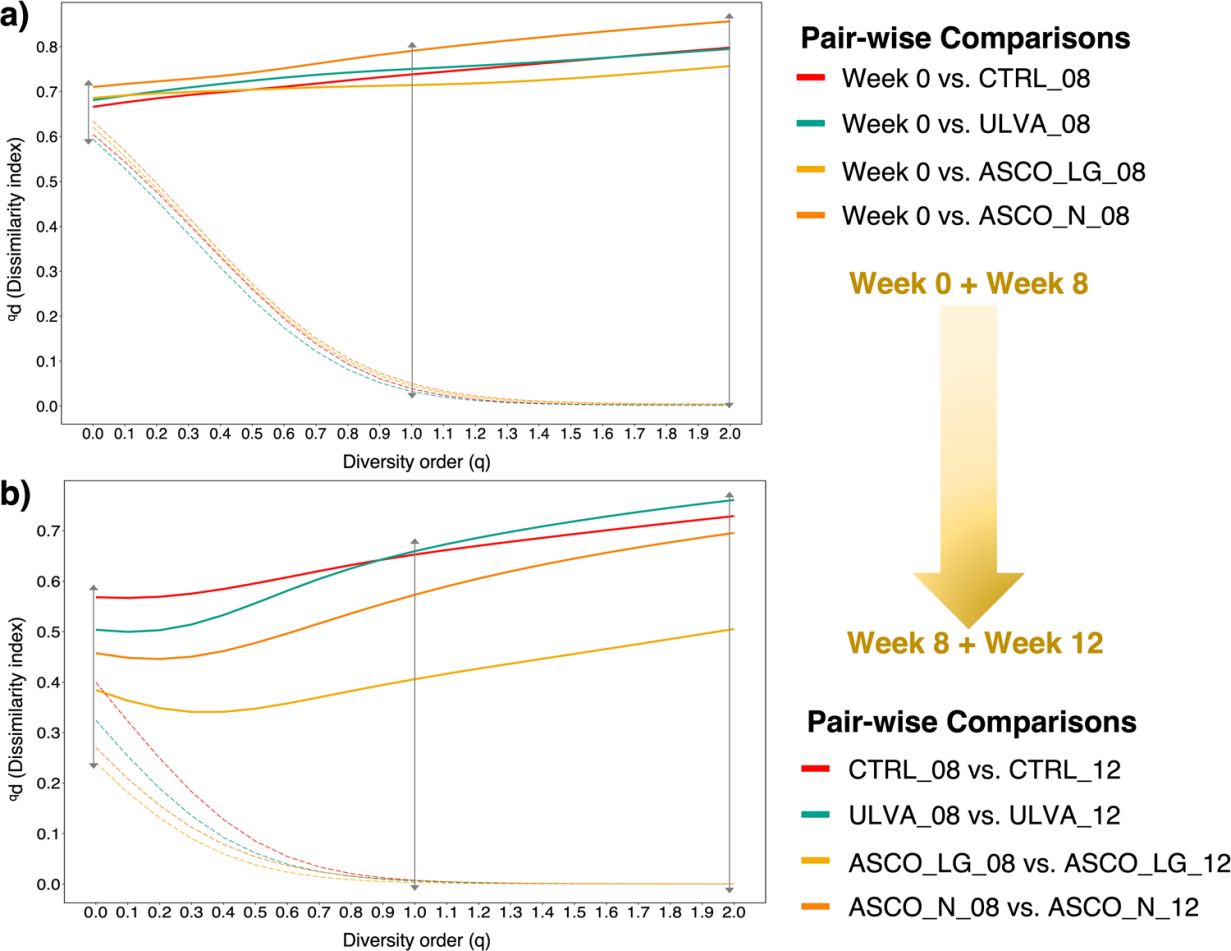
Microbial community assembly in the gut microbiota of Atlantic cod. The presence/absence of OTUs from Week 0 to Week 8 and Week 8 to Week 12 treatments are shaped by random microbial community assembly (at q = 0; empirical values are close to the null expectation). When we increase the importance of OTU relative abundance then assembly is shaped by deterministic processes (q ≥ 1; empirical values far greater than the null expectation). The solid lines show hill-based dissimilarity (qd) and the null expectation is shown by dashed lines. The diversity order (q) curve can be interpreted as follows; q = 0 considers OTU presence/absence, q = 1 OTUs are weighted according to relative abundance, and at q > 1 = increased weighting is given to highly abundant OTUs. Arrows at q = 0, 1, and 2 reflect the distances between the empirical dissimilarity observations and the null expectation at these key points in the diversity order curve (q). Colours reflect the pair-wise comparison tested. **a** shows comparisons between Week 0 and Week 8, and **b** shows comparisons between Week 8 and Week 12.

Now that we have established the shift in microbial community dynamics over time, we wanted to determine what assembly mechanisms could drive this change. First, we used Hill-based dissimilarity indices combined with a Raup-Crick null model^37–39^ to determine the nature of assembly patterns in pair-wise temporal comparisons (stochastic or deterministic assembly). The temporal dissimilarity observed above was corroborated here, with high dissimilarity (^*q*^*d* values > 0.65) between Week 0 and each Week 8 treatment (Fig. 2a). Dissimilarity was lower between the Week 8 and Week 12 comparisons (CTRL and ULVA) but remained high. However, the ASCO_N and ASCO_LG groups showed less dissimilarity over time with ^*q*^*d* values < 0.5 between Week 8 and Week 12, as compared to the other temporal comparisons. When we consider how the empirical dissimilarity values compare to the null expectation, we observe at a diversity order of 0 (q = 0) that values are close to the null expectation and therefore shaped by random assembly. While, at higher diversity orders (q ≥ 1), empirical dissimilarity values differ greatly from the null expectation and are therefore shaped by deterministic forces.

**Fig. 3:**
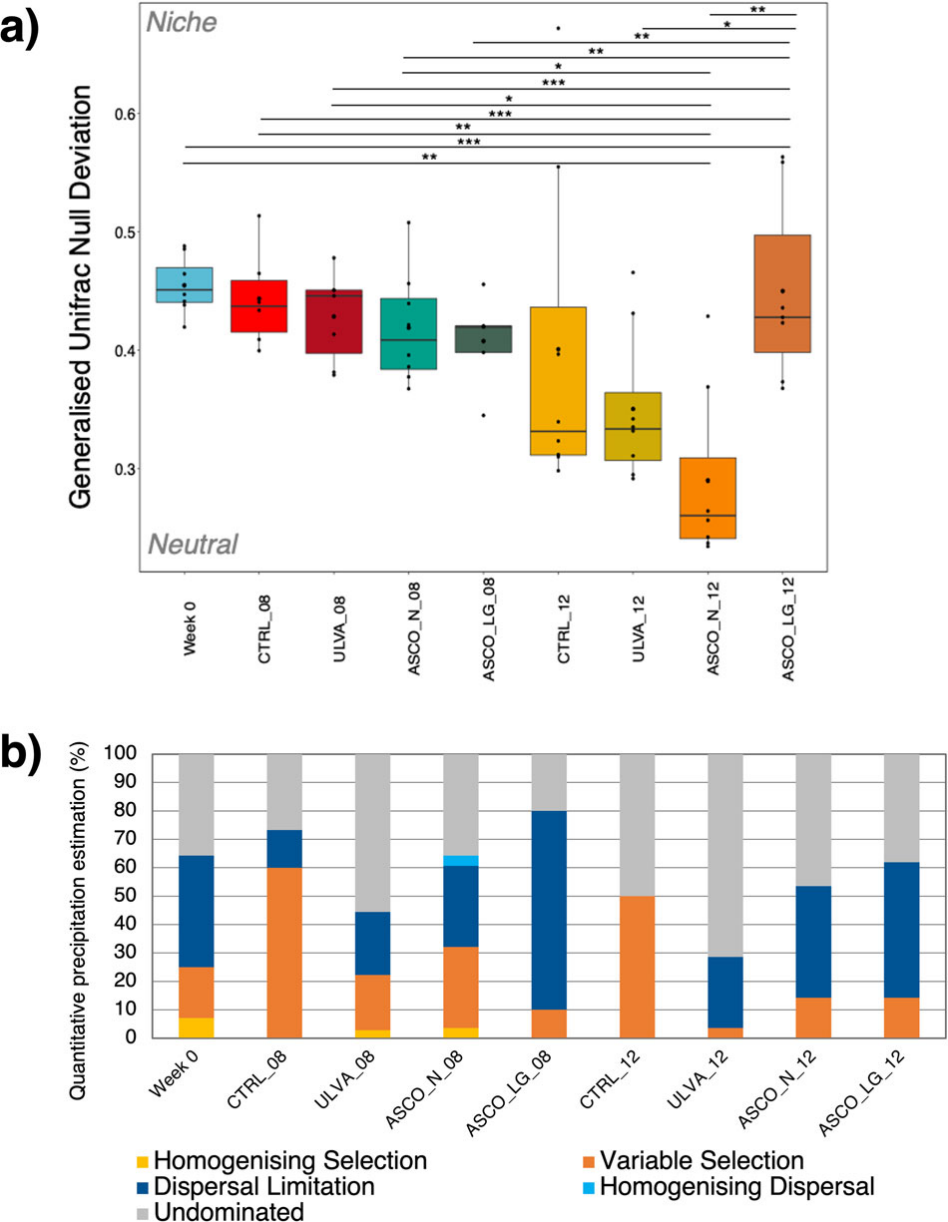
Quantification of microbial community assembly in the gut microbiota of Atlantic cod. **a** The hindgut microbiota of juvenile Atlantic cod is increasingly shaped by neutral assembly processes over time irrespective of dietary treatment, except for the ASCO_LG samples. In the boxplots, center value lines indicate the median, boxes indicate the lower/upper quartiles (25%/75%) and lines extending parallel from the boxes (whiskers) show the variability outside the upper and lower quartiles. Lines connecting categories shows significant relationships (pairwise t-test) with *(*p* < 0.05), **(*p* < 0.01), or ***(*p* < 0.001). **b** The percentage contribution of assembly processes varies over time and across dietary treatments. Variable selection increased in the CTRL samples. Dispersal limitation increased in both ASCO treatments. In comparison, the ULVA treatment showed an increased contribution of undominated processes.

Subsequently, we implemented the beta-null deviation model^34,40^ considering both abundances and phylogenetic distance (Generalised Unifrac dissimilarity measure). We found that the hindgut microbiota showed an increasing contribution of neutral assembly processes (Fig. 3a). Week 12 treatment groups were significantly more neutral than Week 0 (p < 0.05), except for the ASCO_LG samples, which showed an increase towards niche assembly processes over time. Next, we applied the quantitative process estimates measure^41,42^, which assesses the percentage contribution of various assembly processes within treatment groups. At Week 0, dispersal limitation (limited dispersal or historical contingency), variable selection (diverging communities), and homogenising selection (converging communities) accounted for 39, 18, and 7%, respectively, of the assembly processes (Fig. 3b). Undominated assembly (i.e., moderate dispersal and weak selection) accounted for 36% at Week 0. In the control group [CTRL], variable selection increased to 60% at Week 8. At Week 12, variable selection and undominated equated 50% contribution to community assembly in the CTRL treatments. In contrast, the macroalgal diet treatments showed substantial dispersal limitation (25–48%), 40–70% undominated process (weak selection and moderate drift) and low variable selection (4–14%) at Week 12. This was particularly apparent in the ASCO_LG fish, where dispersal limitation accounted for 70% of community assembly at Week 8 and 48% at Week 12. Homogenising selection and homogenising dispersal were not critical assembly processes in the cod hindgut microbiome observed here.

### How do priority effects (competitive lottery model), and species succession (phylogenetic recruitment) effects influence the juvenile Atlantic cod (*Gadus morhua*) microbiome?

There were ten clades (groups of taxa) within the cod hindgut microbiome that had OTUs, which were “lottery winners” (>90% abundance of that clade)—Table 1. According to the threshold described by Verster and Borenstein^30^, four clades displayed strong “lottery-like” assembly behaviour with winners were observed in nearly all samples (winner prevalence >0.75 and winner diversity >0.25). These clades were *Alistipes*, *Cetobacterium*, *Fusobacterium*, and *Tyzzerella* genera (Table 1). Over time, this behaviour changed with both winner prevalence and winner diversity decreasing. For example, the *Alistipes* clade with high prevalence (>0.75) and high diversity (>0.5) at Week 0, decreased from three OTUs (OTU_34, OTU_20, and OTU_3429) to a single OTU at Week 8 (OTU_3429) and a different single OTU at Week 12 (OTU_62). The *Bacteroides* clade showed near lottery-like status at Week 0 with a high prevalence (>0.75) and diversity of 0.2 at Week 0, winner diversity decreased from two OTUs (OTU_9 and OTU_2969) to one OTU at Week 12 (OTU_9). In contrast, *Rikenella* and *Lachnoclostridium* clades (with >50 prevalence) showed low diversity initially with a single OTU winning (i.e., OTU_41 *Rikenella* sp. and OTU_2624 *Lachnoclostridium* sp.). The diversity of winning OTUs increased over time with the addition of an OTU per clade (i.e., OTU_37 *Rikenella* sp. and OTU_130 *Lachnoclostridium* sp.) at Week 12. Many of the remaining clades showed low diversity, with a single OTU indicated as a ‘winner’. There was an overlap between the taxonomy of OTUs that best correlated with the temporal community dissimilarities (Fig. 1) and lottery winners. *Tyzzerella*, *Lachnoclostridium*, *Bacteroides*, *Rikenella* species (Week 8 and Week 12) had lottery-like behaviour (Table 1), while *Photobacterium* or *Vibrio* species did not conform to the lottery schema.

**Table 1:** Genera with operational taxonomic units (OTUs) showing lottery-like behaviour in the hindgut microbiota of Atlantic cod.

Time	Week 0		Week 8		Week 12	
Genus	Prevalence	Diversity	Prevalence	Diversity	Prevalence	Diversity
*Bacteroides*	0.75	0.231	0.466	0	0.625	0
	OTU_9 + OTU_2969		OTU_9		OTU_9	
*Rikenella*	0.60	0	0.461	0.579	0.562	0.317
	OTU_41		OTU_41 + OTU_37		OTU_41 + OTU_37	
*Tyzzerella*	0.857	0.325	0.666	0.272	0.8	0.207
	OTU_23 + OTU_153		OTU_23 + OTU_153		OTU_23 + OTU_90	
*Macellibacteroides*	0.333	0	0.454	0.613	0.067	0
	OTU_2550		OTU_982 + OTU_2550		OTU_2550	
*Fusobacterium*	0.75	0.410	0.385	0.455	0.071	0
	OTU_3155 + OTU_2525		OTU_2525 + OTU_3155		OTU_2525	
*Lachnoclostridium*	0.857	0	0.461	0.325	0.437	0
	OTU_2624		OTU_2624 + OTU_130		OTU_2624	
*Alistipes*	0.428	0	0.231	0.683	0.066	0
	OTU_34		OTU_34 + OTU_3429 + OTU_20		OTU_62	
*Cetobacterium*	1	0.406	0.5	0.361	0.357	0.485
	OTU_2113 + OTU_355		OTU_355 + OTU_2113		OTU_3117 + OTU_355	
*[Anaerorhabdus] furcosa group*	0.043	0	0.2	0	0.333	0.454
	OTU_3118		OTU_3118		OTU_3118 + OTU_3110	
*Erysipelatoclostridium*	0.25	0	0.143	0	–	–
	OTU_3351		OTU_2180		–	

The columns show the winner prevalence (percentage abundance of lottery winners across samples), winner diversity (count of winning OTUs within a genus across samples) and OTUs identified from Week 0, Week 8, and Week 12 for each genus.

**Fig. 4:**
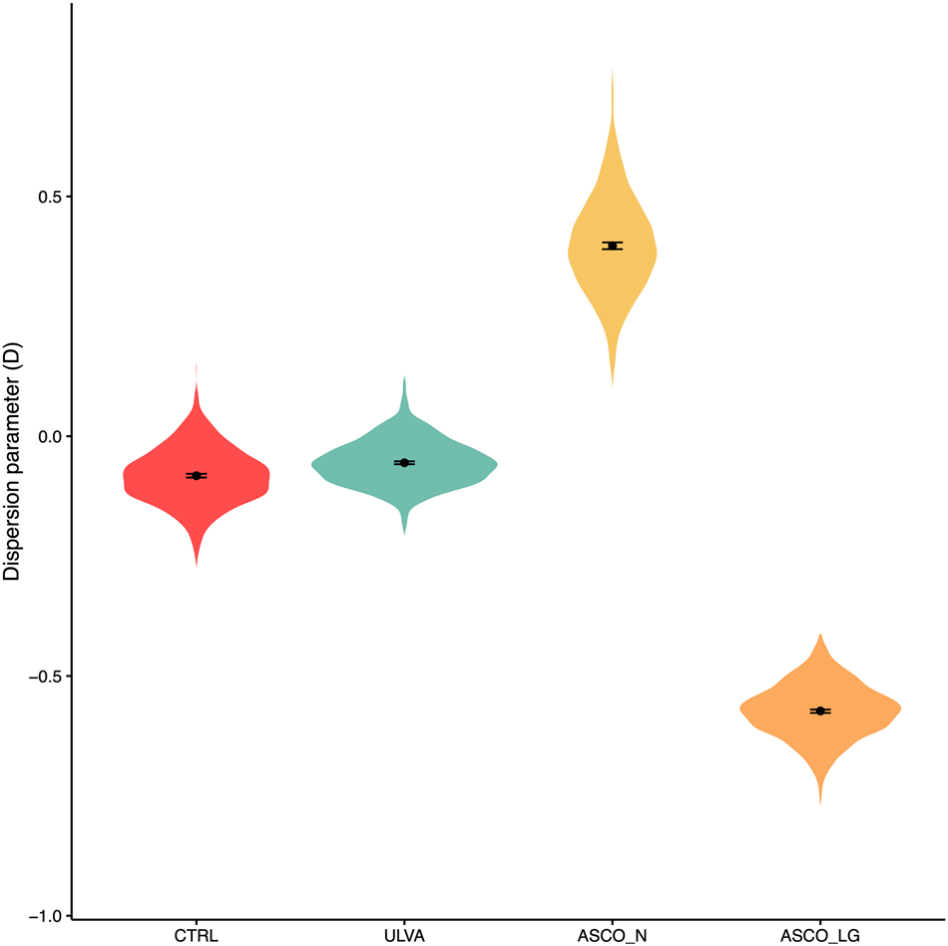
Microbial species succession in the gut microbiota of juvenile Atlantic cod. The hindgut microbiome of juvenile Atlantic cod fed that ASCO diet showed temporal phylogenetic underdispersion (ASCO_LG) and temporal phylogenetic overdispersion (ASCO_N). The CTRL and ULVA treatments varied from slightly neutral to underdispersed. Each violin plot shows the distribution of Dispersion estimates (D) given by logistic error model bootstraps. We have used a 95% bootstrapped confidence interval (with 2000 bootstraps), as shown by the error bars within the plot.

We implemented the phylogenetic recruitment model to examine the temporal assembly trends in species succession. The hindgut microbial communities from the CTRL and ULVA dietary fish groups were slightly neutral tending towards phylogenetic underdispersion with values of D > 0 or <0 (Fig. 4). In contrast, in the hindgut of the ASCO_N fish species recruitment showed phylogenetic overdispersion (D > 0; D = ~0.45), while species recruitment within the ASCO_LG group showed strong phylogenetic underdispersion (D < 0; D = ~−0.55). This suggests that the ASCO_LG group is relatively more nepotistic than the other categories considered.

### How stable is the hindgut microbiome to taxonomic perturbation?

**Fig. 5:**
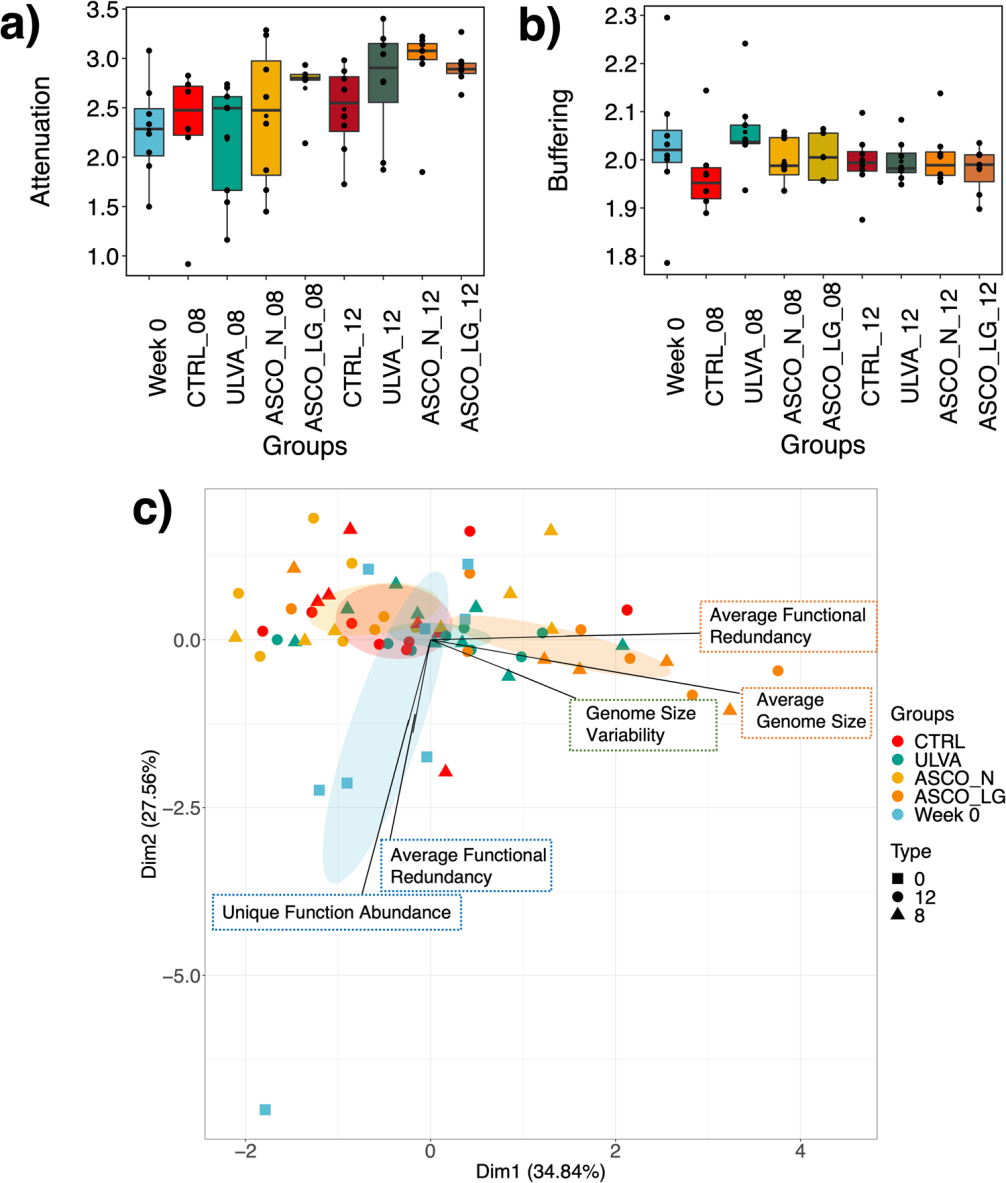
How robust is the gut microbiota of Atlantic cod to taxonomic perturbation? **a** Potential robustness to perturbation magnitude (attenuation) increased over time in the hindgut of juvenile Atlantic cod, with highest values in the ASCO_LG dietary treatment. In the boxplots, center value lines indicate the median, boxes indicate the lower/upper quartiles (25%/75%) and lines extending parallel from the boxes (whiskers) show the variability outside the upper and lower quartiles. Lines connecting categories shows significant relationships (pairwise t-test) with *(*p* < 0.05), **(*p* < 0.01), or ***(*p* < 0.001). **b** Potential buffering capacity towards functional perturbation was relatively stable over time and across treatment groups. In the boxplots, center value lines indicate the median, boxes indicate the lower/upper quartiles (25%/75%) and lines extending parallel from the boxes (whiskers) show the variability outside the upper and lower quartiles. Lines connecting categories shows significant relationships (pairwise t-test) with *(*p* < 0.05), **(*p* < 0.01), or ***(*p* < 0.001). **c** The microbial communities in the hindgut of juvenile Atlantic cod were separated according to variation in gene distribution features (GDF) between Week 0 and Week 8/12 macroalgal dietary treatments. The points represent grouped samples with ‘Time’ denoted by symbols and dietary treatments denoted by colours; Week 0 (blue), CTRL (red), ULVA (green), ASCO_N (light orange), ASCO_LG (dark orange). The percent variance explained by each principal component is indicated on the axis labels. The line and box with the GDF name show the direction of the GDF vectors.

Finally, we used the taxa-function robustness measure to determine the robustness of the cod hindgut microbiome to perturbation. This considers two important values; Attenuation—*high attenuation indicates strong robustness due to functional overlap* and Buffering—*high values indicate functions are balanced across the communities*. Attenuation values increased over time from 2.2 at Week 0 to 2.7 at Week 12. Attenuation was highest in the ASCO treatments at Week 12 (Fig. 5a). Treatments at Week 8 and Week 12 were significantly different (Supplementary Table 1: Tukey HSD *p* < 0.005). Buffering values were in the range of 1.9–2.05 and no temporal or treatment effects were noted (Fig. 5b). Eng and Borenstein^43^ identified five key gene distribution features (GDF) that can account for differences in robustness. When we implemented this measure to compare time and treatment groups, Week 0 microbial communities were clustered towards increased unique function abundance and higher average functional redundancy (Fig. 5c). The ASCO_LG Week 8 and Week 12 samples, and to some extent the ULVA samples clustered towards increased average genome size, genome size variability, and average functional dissimilarity (Fig. 5c). Note, we have performed this analysis again comparing Week 0, Week 8, and Week 12 groups (excluding ASCO samples) and observed a similar pattern (Supplementary Fig. 2).

**Fig. 6:**
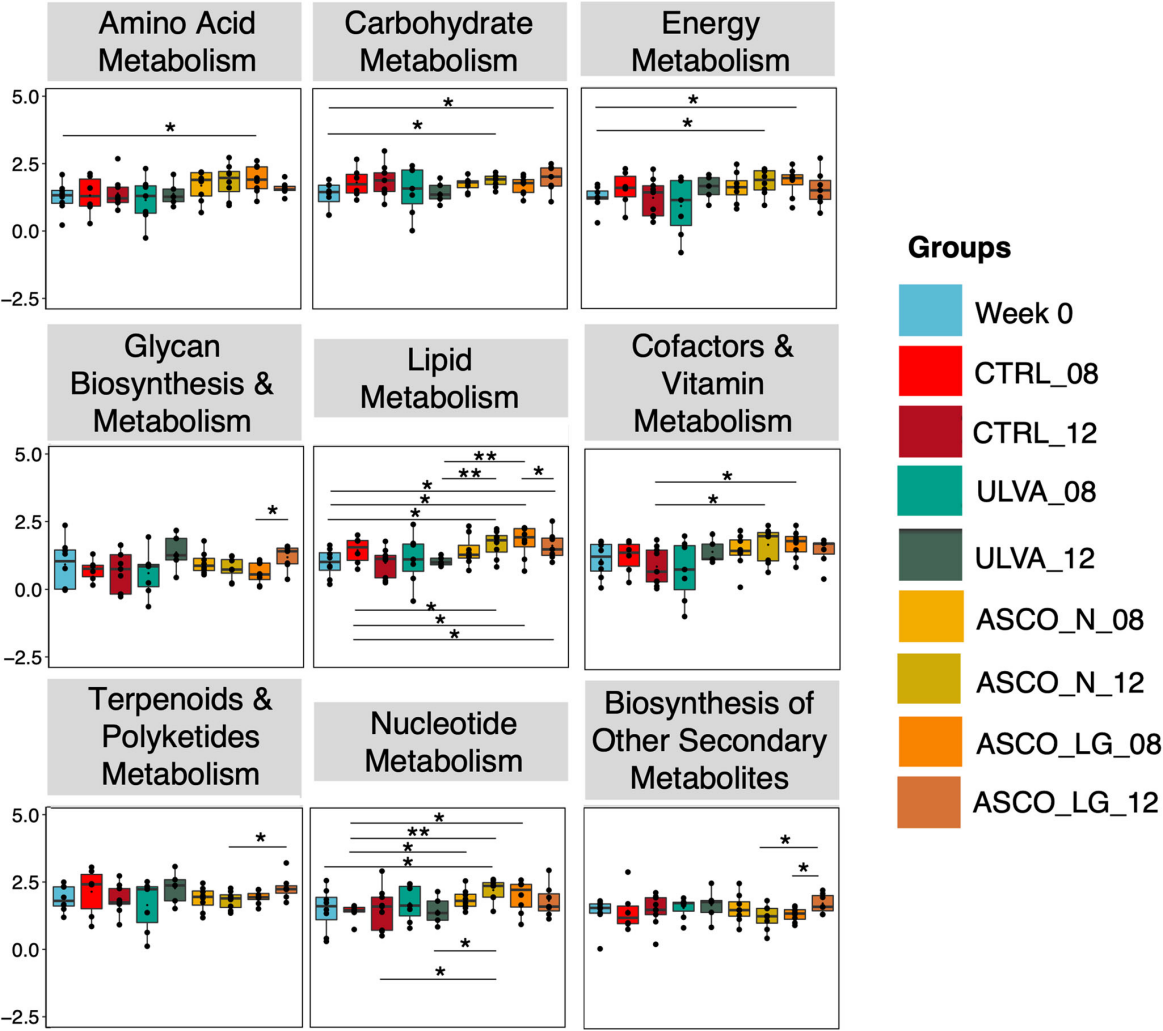
How robust are specific functions in the gut microbiota of Atlantic cod? Potential robustness to specific functional superpathways related to metabolism and cell function were significantly increased over time. In the boxplots, center value lines indicate the median, boxes indicate the lower/upper quartiles (25%/75%) and lines extending parallel from the boxes (whiskers) show the variability outside the upper and lower quartiles. Lines connecting categories shows significant relationships (pairwise t-test) with *(*p* < 0.05), **(*p* < 0.01), or ***(*p* < 0.001).

In general, the robustness (attenuation) of many specific functions increased over time from Week 0 to Week 12 (Supplementary Fig. 3). Functions were categorised into superpathways relating to metabolism and cell function. For example, the attenuation values of carbohydrate metabolism increased from ~1.0 at Week 0 to 2.2 at Week 12 (Supplementary Fig. 3). Focusing on the metabolism superpathways, when we compared metabolism function robustness across dietary treatments, the ASCO treatments were typically increased (Fig. 6). The dietary subgroup ASCO_LG had increased values for functional superpathways related to the excretory system, translation, transport, and catabolism (Supplementary Fig. 4). In contrast, the attenuation values for functional superpathways related to infectious diseases, cell growth and death, replication and repair increased over time, in all but the ASCO_LG samples (Supplementary Fig. 4). The buffering values for superpathway functions related to metabolism (metabolism of cofactors and vitamins, biosynthesis of other secondary metabolites) decreased over time and across all treatment groups, except ASCO_LG (Supplementary Fig. 5).

## Discussion

The fish gut microbiome is essential to systemic function, fish health^44^, immune support^45^, and digestive capacity^46^. However, the factors that govern the microbial community colonisation, assembly, and succession in fish species are poorly understood, in terms of both the ecological assembly mechanisms and development within the life cycle. Indeed, in our previous work^21^, we noted a shift in the hindgut microbial community dynamics of juvenile Atlantic cod over time. This shift was summarised in the current manuscript as a displacement in Proteobacteria (mostly *Photobacterium* spp.) with Bacteroidetes and Firmicutes species. Interestingly, this pattern was not observed in fish that found one of the experimental diets unpalatable (ASCO_LG). In this study, we endeavoured to determine the ecological processes that may shed light on this temporal convergence in the hindgut microbiome. We implemented a suite of ‘null-modelling’ approaches to determine what assembly processes were driving the microbial community dynamics in the hindgut of juvenile Atlantic cod. We then used the taxa-function robustness measure to assess the potential stability of the gut microbiome functions to taxonomic perturbation. Understanding such mechanisms is an important step towards managing the response to newly introduced aquafeed ingredients, improving welfare in commercial facilities or indeed conducting targeted manipulations of the microbiome for improved fish health.

We showed high temporal dissimilarity within the hindgut of juvenile Atlantic cod using the Hill-based dissimilarity and null model methodology of Modin et al.^37^. Interestingly, we further revealed that dissimilarity with respect to the presence or absence of OTUs was driven by random (stochastic forces) such as births, deaths or ecological drift^34^. However, we found that deterministic forces were driving dissimilarity in the abundances of OTUs (diversity order q > 1). Deterministic assembly processes include those which are shaped by environmental conditions (e.g., pH, nutrient availability), species interactions (e.g., competitive exclusion), or species traits (species genetics). Thus, in the cod hindgut microbiome, we observed dissimilarity in the abundance of common OTUs over time, which may be explained by selective-type pressures/conditions favouring the proliferation of particular species. This finding agrees with our hypothesis that the gut microbiome would demonstrate deterministic assembly. Deterministic factors here could also include selective pressures from the host, particularly during phases of development^47^. The gut microbiota of Atlantic cod within the larval phase is driven by selective pressures, associated with host intestinal development combined with some stochastic pressures^48,49^. Similar results were observed in Gibel Carp (*Carassius auratus gibelio*)^50^ and zebrafish (*Danio rerio*)^47,51^. The zebrafish studies notably cover the complete lifecycle (which is more practical given their shorter lifecycle). In our current study, fish were 366 days post-hatch, and it is estimated that wild Atlantic cod (from the Irish Sea) mature within two or three years of hatching^52^. Host pressures in the juvenile or adult cod gut are not widely described, a study design similar to that of Xiao et al.^47^ taking into account the complete life cycle of Atlantic cod could help elucidate these factors. The addition of proteomics to characterise the Atlantic cod gut responses during development would enable direct linkage of the microbiome to the host response.

Contrary to our hypothesis, we also observed a temporal trend from niche to neutral processes. This is consistent with Hayes et al.^53^, where the authors showed neutral processes dominated the gut microbiome in farmed Atlantic salmon (*Salmo salar*). Multiple processes can occur in a system given the complexity of the biology and interactions with the environment^54^. Indeed, the gut environment may add a first-pass deterministic pressure as only species capable of withstanding the gut conditions will proliferate over time^55,56^. This theory is perhaps further evidenced by the results from the hill-based method, where we found the presence of OTUs was driven by stochastic mechanisms, but abundances were deterministic in nature. We further quantified the contribution of different ecological processes. We discovered that dispersal limitation, variable selection and undominated (weak selection and moderate dispersal) were the primary ecological processes occurring in the cod gut microbiome. The contribution of these processes varied with respect to time and treatment. For example, variable selection increased at Week 8 for all groups and was the control group’s primary process at Week 12. Variable selection indicates strong selective pressures driving divergent shifts in species composition. However, variable selection was not a dominant process in the macroalgae treatments at Week 12. Yet, the communities in the CTRL, ULVA, and ASCO_N converged in taxonomic composition over the trial without evidence of homogenising selection.

In the macroalgal dietary treatments dispersal limitation was a core process in the hindgut, this effect was increased in the ASCO_LG fish that found the diet unpalatable. We have not directly cross-compared treatment groups, as each sample represents an individual fish, treatment groups were in separate tanks, and we had sacrificial temporal sampling thus direct dispersal would have been limited between these communities. As defined by Stegen et al.^42^, dispersal limitation indicates that a low dispersal rate is the primary cause of high compositional turnover (aiding ecological drift or stochastic processes), although the framework does not account for in situ diversification. This would occur where dispersal rates are low and new OTUs that evolve within a single taxon may only be present in one community. Given the gut structure whereby the digesta content is contained within the digestive tract (i.e., a semi-closed system), of individual fish, diversification could be an uncharacterised influence here.

The digestive system is a space-limited system, stochastic models such as the competitive lottery model can shed light on what species will be first to occupy the niche space and thus, manipulate the conditions for subsequent species. Clades that showed very strong lottery-like behaviour (*Alistipes*, *Cetobacterium*, *Tyzzerella*, and *Fusobacterium*), primarily only did so at Week 0. This indicated that there was a temporal effect and/or that the physical change from a commercial diet to in-house pellets may have impacted microbial competition for space in the hindgut. To fully elucidate this, earlier time points including the former diet would be required (Experiment started Day 366 post-hatch baseline before changing feed—Week 0). We observed overlap between the genera that correlated with the temporal community dissimilarities at Week 8 and Week 12 and lottery winners (*Tyzzerella*, *Lachnoclostridium*, *Bacteroides*, *Rikenella*) species. While *Photobacterium* (Week 0), which decreased over the course of the trial did not conform to the lottery schema. *Photobacterium* species have a high prevalence in many fish species, including wild cod populations^23,57^. Notably, *Photobacterium* was completely absent from lottery winners (i.e., no OTUs were > 90% within this clade in our samples). Species that do not conform to the competitive lottery schema are thought to be less specialised in their niche and co-exist with other species^30^, which would support the widespread distribution of *Photobacterium* across fish species^19^. This result aligns with the gene distribution features showing high functional redundancy at Week 0, whereby multiple taxa can carry out the same function. The prevalence of lottery winners (*Bacteroidetes* and *Firmicutes*) increased at Week 8/Week 12 (low functional redundancy/increased unique function). Members of the *Bacteroidetes* phylum, have been highlighted as highly flexible generalists and specialists^58^. Authors have noted that the gut microbiome in wild Atlantic cod had limited diversity and exhibited phylogenetic nepotism^59^. We noted similar, and this was particularly apparent in the ASCO_LG fish. In most of the tested community assembly methods, the ASCO_LG fish did not follow the same trend as the other dietary treatments, showing, for example, temporal trends towards strong phylogenetic underdispersion (more nepotism) and niche assembly. Thus, indicating that microbial community assembly patterns were disrupted in fish that displayed poorer growth rates and found the diet unpalatable. These microbial community assembly patterns may drive unexpected changes in fish condition and function^60^. This indicates that diet selection could play a role in the disruption of the microbiome development in farmed fish^61^.

The gut microbiome of any species is integral to the functioning of the system, microbial imbalance can lead to harmful impacts to the host in a condition called dysbiosis^62^. This occurs when the microbial community changes result in a change in function, leading to altered pathways, and the production of excess acids for example. However, the microbial taxonomic composition of a system can fluctuate without detectable changes to the inherent functioning. We observed taxonomic shifts in the hindgut microbial community over time, and changes in the predicted functional pathways. This may have been related to community imbalance or a natural succession of the communities. In gut microbial communities, the relationship between species taxonomic and functional profiles has been defined as the ‘taxa-function relationship’. This relationship can be viewed as a landscape containing the breadth of microbial taxa and inter-related functional capacities. Using such a landscape, the situations resulting in dysbiosis (microbial imbalance which is harmful to the host) can be assessed. We used the *taxa-function robustness* measure^43^ to calculate the breadth of taxonomic shifts (perturbations) and the community functional capacity. In our work, we showed that in the hindgut Atlantic cod microbiome, the microbial communities increased in robustness and functional stability over time, despite temporal taxonomic shifts. Robustness was particularly increased for functions involved in metabolism and cell regulation. Increased robustness in metabolism-based function and cell cycle functions, particularly in the ASCO treatments needs further investigation beyond predictive functions, as we speculate that this result would not necessarily correlate with increased resilience to disturbance. Functional analysis of microbial communities in complex systems, such as the gut has a greater utility than taxonomic profiles^63^.

There are some potential limitations to our study. In our analysis, we have not considered the source metacommunity (tank seawater). While research has suggested that changes in the gut microbiome of Atlantic cod larvae^49^ and common molly adults (*Poecilia sphenops*)^64^ can occur independently of the tank rearing water, it is an unaccounted-for variable in our present study. Authors have used changes in tank water to manipulate the microbiome of Atlantic cod larvae using ecological theory to improve survival rates using selection^65^. To use these tools in a controlled and predictable manner we must first unravel what assembly processes are occurring and how we can exploit the microbial interactions. Moreover, targeted manipulations of the gut microbiome with aquafeed ingredients, like probiotics, must consider the interaction between adherent and non-adherent gut microbial communities^66^, an aspect not explored in this study. Resolving these issues may offer an opportunity to use the gut microbiome to develop fish with the improved condition and immune competence for commercial use as in aquaculture or in wild population restocking programmes.

**Fig. 7:**
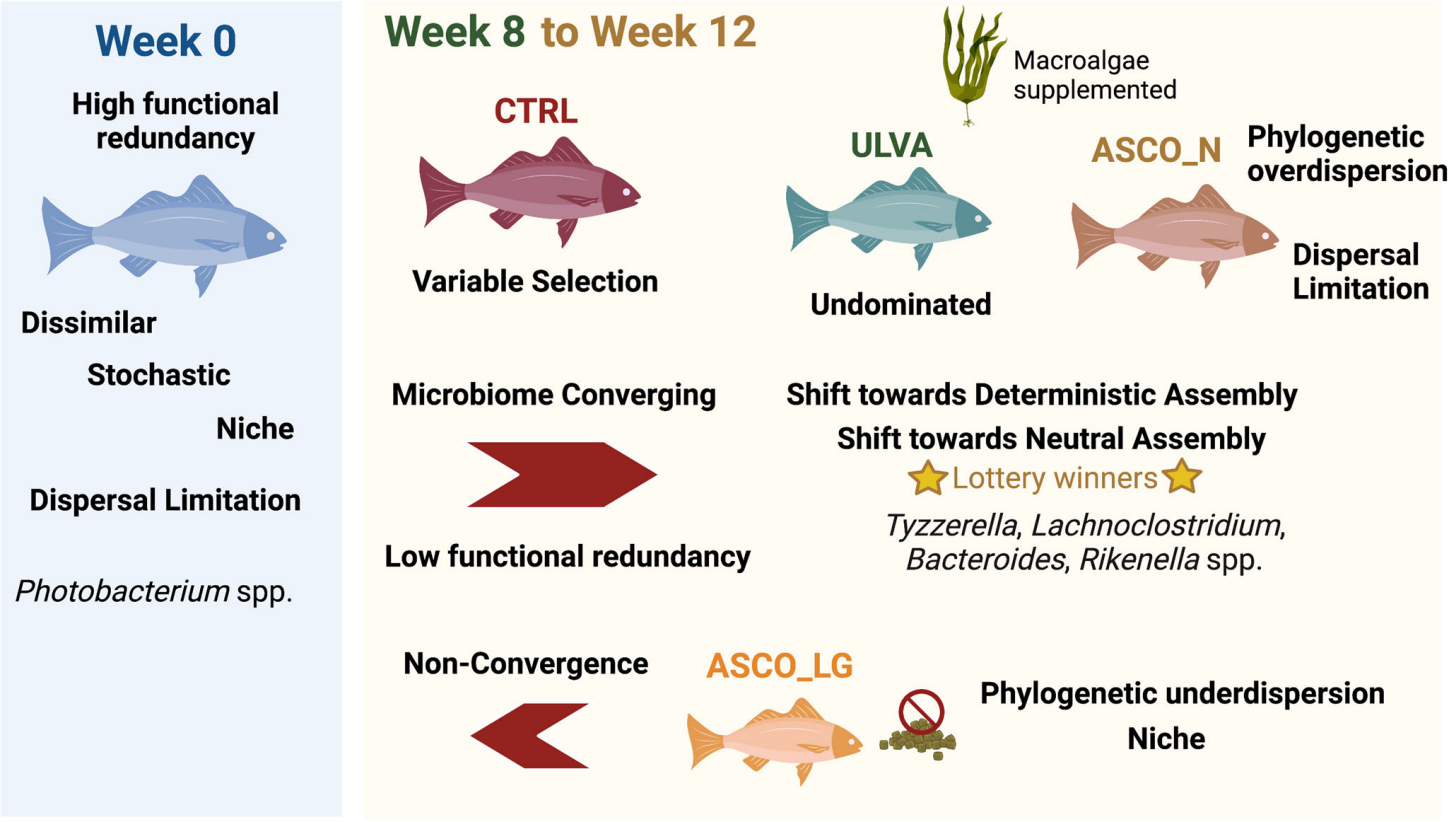
Summary of our key findings on microbial assembly in the Atlantic cod hindgut. The schematic figure was created with Biorender.com and shows the changes in community and assembly over time (Week 0, Week 8, and Week 12) and across treatments (Week 0, CTRL, ULVA, ASCO_N, and ASCO_LG).

In summary, the ecological drivers of microbial community assembly in the gut microbiome are important factors to consider when linking microbial community composition and diversity to fish health and environmental parameters. We conclude that the microbiome in the gut of Atlantic cod (*G. morhua*) in an experimental feeding trial over a twelve-week period was under the influence of multiple assembly processes; stochastic forces shaping the presence or absence of OTUs, deterministic forces and a trend from niche to neutral processes. We quantified these processes as an increase in variable selection in the control diet (divergence in communities related to selective environmental conditions) over time. Dispersal limitation was a driver of the gut microbiome for fish fed the macroalgae supplemented diet at Week 12. Generalist *Photobacterium* species (Week 0) decreased over the course of the trial and were absent from the lottery model. There was overlap between the genera that were increasing over time and lottery winners (*Tyzzerella*, *Lachnoclostridium*, *Bacteroides*, *Rikenella*) species at Week 8/Week 12. This corresponded to a shift from high to low functional redundancy. The recruitment of new taxa overtime was altered in the ASCO groups (10% *Ascophyllum nodosum* supplement), with individuals who found the diet unpalatable exhibiting phylogenetic underdispersion (nepotistic recruitment of species). The ecological processes are summarised in Fig. 7. Finally, the gut microbiome showed increasing robustness to taxonomic disturbance over time, which was increased in the ASCO_LG individuals. These fish showed an altered microbiome, with increased susceptibility to functions related to infectious diseases and cell regulation and increased robustness of metabolism functions. Although our study focused on the juvenile cod gut microbiome, many of these findings are of broad interest in fish research, and indeed to the wider field of gut microbiome research. We highlight research areas that warrant further investigation in fish farming trials; (i) how the host influences ecological assembly over the complete life cycle of Atlantic cod (*G. morhua*), (ii) increased investigation into gut functional pathways, particularly in response to stress and (iii) microbial network analysis to elucidate the interactions between source water, feed source, adherent, and non-adherent bacteria. These should be subsequently followed up with targeted and informed microbial manipulation experiments. This invaluable information would allow for better animal health management (for Atlantic cod in particular), to increase the resilience of farmed species, particularly towards the use of functional feed additives, such as prebiotics and probiotics for improved gut health.

## Methods

Juvenile Atlantic cod (*G. morhua*) were hand-graded (123 ± 7 g, SD) and randomly allocated to one of nine experimental tanks (three tanks per treatment and 60 fish per tank). Fish were acclimated for one week on a commercial fishmeal diet (Amber Neptun, Skretting, Stavanger, Norway), noted as the Week 0 sampling phase. Then tanks were assigned to the following diets, a 10% dietary macroalgae supplement (either *Ulva rigida* [ULVA] or *Ascophyllum nodosum* [ASCO] species) or a control diet for basal comparison, i.e., no algal addition [CTRL]. At Week 8 within the feed trial, a subgroup of the ASCO fish displaying ‘reduced growth rates were observed and were likely due to reduced acceptance of the feed^21^. We, therefore, subcategorised this group as [ASCO_LG], and the remaining fish with ‘normal’ growth was referred to as [ASCO_N]. The feed trial was carried out for twelve weeks.

### Sample collection, DNA extraction and 16S rRNA amplicon sequencing

Fish were removed from the tanks at Week 0, Week 8 and Week 12 and euthanised with an overdose of tricaine methanesulphonate solution (MS222, Pharmaq, Overhalla, Norway). The brain was then destroyed to confirm death according to regulations for animal welfare (EU Directive 2010/63/EU). We then removed the last 10–15% of the digestive tract and placed in sterile microcentrifuge tubes. There were 67 fish gut samples arising from eight fish at Week 0, 28 fish at Week 8 (6 CTRL, 9 ULVA, 8 ASCO_N, 5 ASCO_LG) and 31 fish at Week 12 (8 CTRL, 8 ULVA, 8 ASCO_N, 7 ASCO_LG). Variability in sample numbers was due to some fish not having sufficient gut content for eventual analysis. Samples were transported to the National University of Ireland Galway on dry ice and stored at −80 °C. DNA extractions from hindgut digesta per sample were carried out by phenol-chloroform extraction with an additional bead-beating step using Lysing Matrix E tubes (MP Biomedical, Illkirch-Graffenstaden, France)^21,67^. Sample DNA and DNA from a negative extraction control (nuclease-free water, Qiagen, Venlo, The Netherlands) were sent to the Research Technology Support Facility at Michigan State University (Michigan, USA) for sequencing. Amplicon sequencing of the 16 S rRNA gene targeting the V4 hypervariable region was performed using the universal primer set [515 f/806r^68^]. Sequencing was carried out using the Illumina technology using a standard flow cell and 500 cycle v2 reagent cartridge (Illumina Inc., Hayward, California, USA).

### Ethics approval and consent to participate

The feeding trial was carried out in 2014 at the Carna Research Station, Carna, Co. Galway, Ireland, a Health Products Regulatory Authority (HPRA) licensed institution. All personnel involved in the feed trial work were Laboratory Animal Safety Trained Ireland (LAST-Ireland) with individual authorisation as per our previous publication^21^.

### Bioinformatics

The methods to generate all microbial data (e.g., operational taxonomic units (OTUs), phylogenetic trees and biom. files) are given in Supplementary Methods.

### Subset analysis

For finding key microbial species that contribute to beta-diversity between samples over time we used the “BVSTEP” routine^69^. This method reduces the abundance table down to the subset of OTUs which can roughly still explain the same beta-diversity pattern as the full OTU table, therefore finding the key OTUs. Briefly, the method calculates the Bray-Curtis distance between samples using all the OTUs and records it as original distances. It then permutes through the subset of OTUs, and for each permutation, it calculates the Bray-Curtis distances between the samples again and correlates these distances against the original recorded distances until subsets are obtained that explain roughly the same beta diversity as the full set of OTUs. To run this algorithm, bvStep() (from the sinkr package) was used^70^. After obtaining the subset of OTUs, we used R’s ‘Vegan’ package^71^, particularly the bioenv () function to regress the subsets on top of the principal coordinate analysis (PCOA) plot.

### Microbial community assembly

#### Hill-numbers dissimilarity indices (^*q*^*d*)

Hill numbers are a set of indices parameterised by q representing the diversity order which determines the weight given to the relative abundance of OTUs in a community. Modin et al.^37^ derived the beta-diversity equivalent of hill numbers (^*q*^*d*) as a dissimilarity index of diversity order where ^*q*^*d* values are scaled between 0 (similar) and 1 (dissimilar). The authors used this approach to illustrate how OTU abundance contributes to the dissimilarity between communities. Here, we compared ^*q*^*d* at q = 0 (presence/absence, i.e., Jaccard index), q = 1 (OTUs abundances are weighted i.e., Bray-Curtis) and q = >1 (OTUs with greater relative abundance have increased weighting). Further, a randomisation scheme was applied and repeated many times to obtain the null distribution for the dissimilarities across this scale between communities. These null distributions when compared to the observed dissimilarity (^*q*^*d*) reveal ecological insights, i.e., if the values are similar, the observed dissimilarity can be explained by stochastic factors, and if higher or lower than the null expectation, then there are likely deterministic factors that favour different or similar microbial taxa in two categories. These indices and null distributions were calculated using the qdiv Python software^37^.

#### Beta-null model

Beta-null deviation measures were calculated according to Tucker et al. and Lee et al.^34,40^. The method first calculates the pairwise observed dissimilarities (β_obs_) between samples using the Generalised Unifrac dissimilarity measure. By preserving the alpha diversity in the observed samples, random communities are generated (999 randomisations) to calculate the beta diversity measure again for these communities (β_null_) and then the deviation from the observed dissimilarities are recorded. The average of the deviations gives a numerical value that differentiates between niche (values further from 0) and neutrally structured communities (values nearer to 0). We have done this separately for each treatment group on a temporal basis (Week 0, CTRL_08, ULVA_08, ASCO_N_08, ASCO_LG_08, CTRL_12, ULVA_12, ASCO_N_12, and ASCO_LG_12).

#### Quantitative process elements

Quantitative process elements (QPE) were used to assess the influence of ecological processes based on the conceptual framework of Vellend et al.^72^ and implemented according to Stegen et al.^41,42^. This framework provides a quantitative measure of the influence of selection and dispersal pressures on microbial community structure. Selection considers deterministic selective pressure which results in divergent (variable selection), or convergent (homogenous selection) communities often considered over time. While dispersal considers the spatial movement of species where the increased movement of species results in convergent communities (homogenising dispersal), or limited movement of species results in divergent communities through drift (dispersal limitation). The authors also included the category ‘Undominated’ to describe the situation whereby neither selection nor dispersal processes dominate. The framework considers the phylogenetic distance and phylogenetic turnover between closely related OTUs in pairwise samples^28^. This is achieved using the abundance-weighted β-mean-nearest taxon distance (βMNTD)^73^. To determine how this varied from the null expectation, randomisations were employed whereby the abundances and species names were shuffled across phylogenies to provide a null value^41^. This was replicated 999 times to give the null distribution. The deviation between the null distribution and the observed βMNTD value = β-nearest taxon index (βNTI). If the observed βMNTD value is significantly greater (βNTI > 2) or less (βNTI < − 2) than the null expectation, the community is assembled by *Variable or Homogeneous Selection*, respectively. For the remaining with no significant deviation, in the next step, Raup-Crick was used with the inclusion of OTU relative abundance^38^ termed RC_bray_. RC_bray_ values were compared to the null expectation and the resulting deviation determined the influence of dispersal (RC_bray_ > 0.95). Values of RC_bray_ > −0.95 indicate *Homogenising Dispersal* (transport between microbiomes leading to establishment), while values of RC_bray_ > +0.95 indicate *Dispersal Limitation*. In the latter, this may indicate ‘true’ effects of dispersal limitation (i.e., limited transport across microbiomes and stochastic events) and/or historical contingency. In cases where values were <0.95 the communities were ‘*Undominated*’, i.e., not dominated by a sole ecological process (weak selection and moderate drift).

#### Competitive lottery model

We applied the competitive lottery model as outlined in Verster and Borenstein^30^ which is based on the theory of Sale^74^ first proposed for fish populations. The theory is based on the idea that there is competition between colonising species within a niche and only a single species can ‘win’ in the space (strong priority effect). This ‘winner’ is chosen at random (stochastic process) with an analogy drawn to a ‘lottery’. In microbial ecology this scheme determines clade-based assembly, i.e., within a taxonomic group (a genus), we can determine if the group follows lottery-like behaviour and if so what OTUs ‘win’. A winning species/OTU is defined as a clade member with >90% abundance [see Verster and Borenstein^30^ for details on how this threshold was determined]. Then the diversity of lottery winners was calculated using Shannon diversity index of the winners across samples (how often each OTU occurs as a winner in samples where lottery-like behaviour was observed). Diversity was normalised between 0 and 1 to account for differences in lottery winners. Values approaching 0 indicate that a single OTU is dominating that specific genus in all samples, while values approaching 1 indicate an even distribution of winning OTUs within a genus.

#### Phylogenetic recruitment model

The phylogenetic recruitment model^75^ was then used to describe the order in which new species are detected in the cod hindgut microbiome over time. In this model, the Dispersion parameter (D) is calculated based on the probability of detection of new species on temporal scales by fitting a logistic error model on changes in phylogenetic diversity (PD) estimates. As opposed to the previous models, time-series dependency is assumed. The value of D determines the primary recruitment mechanisms, where D = 0 indicates that all species have an equiprobable chance of recruitment. If D > 0 then taxa that are more phylogenetically divergent (to the taxa detected in previous time-points) are preferentially added to the community (overdispersion). In contrast, if D < 0 then taxa that are more phylogenetically similar (to the taxa detected in previous time-points) are preferentially added to the community (underdispersion).

### Functional analysis and taxa-function robustness

#### Functional analysis

The functional potential was obtained as KEGG orthologs (KO) and pathway predictions by using Phylogenetic Investigation of Communities by Reconstruction of Unobserved States (PICRUSt2)^76^ and the Quantitative Insights into Microbial Ecology (QIIME2) plugin^77^ using the parameters --p-hsp-method pic --p-max-nsti 2. To find KEGG enzymes/MetaCyc pathways that are significantly different between different categories, we used DESeqDataSetFromMatrix() function from DESeq2^78^ package with the adjusted P-value significance cut-off of 0.05 and log_2_ fold change cut-off of 2. This function uses a negative binomial general linear model to obtain maximum likelihood estimates for OTUs log fold change between two conditions. Then Bayesian shrinkage is applied to obtain shrunken log-fold changes subsequently employing the Wald test for obtaining significances. For KEGG orthologs that were at least log_2_ fold significant, we used iPath3^79^ to give an overview of KEGG pathways for microbial metabolic function.

#### Taxa-function robustness

Following the procedure of Eng and Borenstein^43^, the *taxa-function robustness* measure of the cod hindgut microbial communities was calculated. The principle of the taxa-function robustness measure is to perturb an individual sample several times (100 perturbations) and then calculate a two-dimensional profile of taxonomic shift versus functional shift. To create the taxonomic profiles, weighted Unifrac dissimilarities were calculated across samples and simulated perturbations. To obtain predicted functional profiles, the authors calculated the KEGG Orthology (KO)roups for the whole green genes database (gg_13_5) along with KO copy numbers provided as a reference database (https://github.com/borenstein-lab/robustness), which can be used if the OTUs follow the green genes nomenclature. For the functional profiles, cosine dissimilarities were calculated across samples and simulated perturbations. After obtaining the taxonomic and functional shifts (both denoted as *t*) for a given sample, a relationship between taxonomic perturbation magnitude and functional profile shift is assumed to behave individually as $f = \frac{1}{{e^a}}t^b$ and fitted using the linear regression model on natural log transformed data: $\ln \left( f \right) = - a + b\ln (t)$. In the equation, *f*. denotes the expected shift in functional profile and we can estimate **a** (termed as “attenuation” coefficient describing *the expected rate at which increases in the taxonomic perturbation magnitude are expected to increase functional profile shifts*) and **b** (termed as “buffering” coefficient indicating *how large a perturbation must be before a functional profile shift becomes noticeable*). The coefficients thus serve as proxies (robustness factors) to summarise the property of a sample to withstand perturbation. These were calculated for all the samples in the dataset. Additionally, the main gene distribution features (GDFs) across the genomes of species in a community, were then displayed as a PCOA plot. Further details can be found in Eng and Borenstein^43^.

### Reporting summary

Further information on research design is available in the Nature Research Reporting Summary linked to this article.

## Supplementary information

Supplementary Information

Reporting Summary

## Data Availability

The raw sequences are available in the SRA database under Bioproject Submission PRJNA636649.
