# Supplementary material for: Drivers of ecological assembly in the hindgut of Atlantic Cod fed a macroalgal supplemented diet
Source: NPJ Biofilms Microbiomes. 2022 May 4;8:36. doi: 10.1038/s41522-022-00296-x (PMC9068720; doi:10.1038/s41522-022-00296-x)
Supplement: Supplementary file 1 — Supplementary Information [file 41522_2022_296_MOESM1_ESM.pdf]

Drivers of ecological assembly in the hindgut of Atlantic Cod fed a macroalgal supplemented diet

C. Keating[1,2,3], M. Bolton-Warberg[4], J. Hinchcliffe[5], R. Davies[6], S. Whelan[4], A. H. L. Wan[7,8], R. D. Fitzgerald[4], S. J. Davies[9], C. J. Smith[1,2], U. Z. Ijaz[2#]

[1]Microbiology Discipline, School of Natural Sciences, National University of Ireland Galway, Ireland, H91 TK33.
[2]Water and Environment Group, Infrastructure and Environment Division, James Watt School of Engineering, University of Glasgow, Glasgow, United Kingdom, G12 8LT.
[3]Institute of Biodiversity, Animal Health & Comparative Medicine, University of Glasgow, Glasgow G12 8QQ, UK
[4]Carna Research Station, Ryan Institute, National University of Ireland Galway, Carna, Co. Galway, Ireland, H91 V8Y1.
[5]Department of Biological and Environmental Sciences, University of Gothenburg, Gothenburg, Sweden.
[6]AquaBioTech Group, Central Complex, Naggar Street, Targa Gap, Mosta, MST 1761, Malta G.C.
[7]Irish Seaweed Research Group, Ryan Institute and School of Natural Sciences, National University of Ireland Galway, Ireland, H91 TK33.
[8]Aquaculture and Nutrition Research Unit, Carna Research Station, Ryan Institute and School of Natural Sciences, National University of Ireland Galway, Carna, Co. Galway, Ireland, H91 V8Y1.
[9]Department of Animal Production, Welfare and Veterinary Science, Harper Adams University, Newport, Shropshire, UK, TF10 8NB.

[#] Corresponding Author: Umer.Ijaz@glasgow.ac.uk

1. Supplementary Information

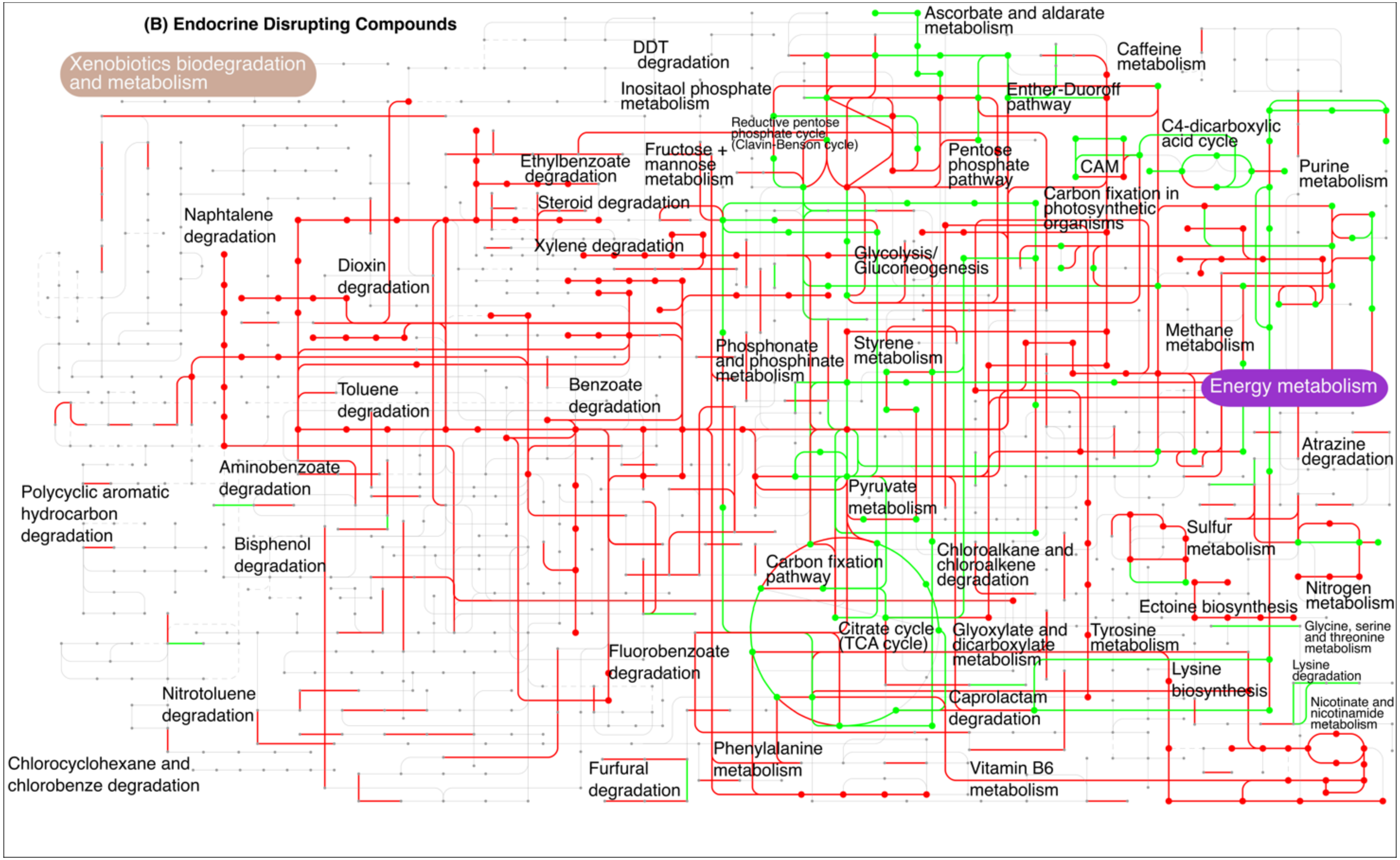

Supplementary Figure 1. Temporal functional shifts in the hindgut of Atlantic cod.

Predictive functional capability of microbial communities in the hindgut of juvenile Atlantic cod changed over time with pathways relating to metabolism pathways increased at Week 12 as compared to Week 0. KEGG orthologs and pathway predictions from the 16S rRNA gene sequencing of the gut microbiome from juvenile Atlantic cod using PICRUSt2 predictive functions[69]. The figure shows the KEGG pathways drawn in iPath3[72] which are significantly different at a $\log_2$ fold change using DeSeq2 ($p < 0.05$) from Week 0 (red) and Week 12 (green).

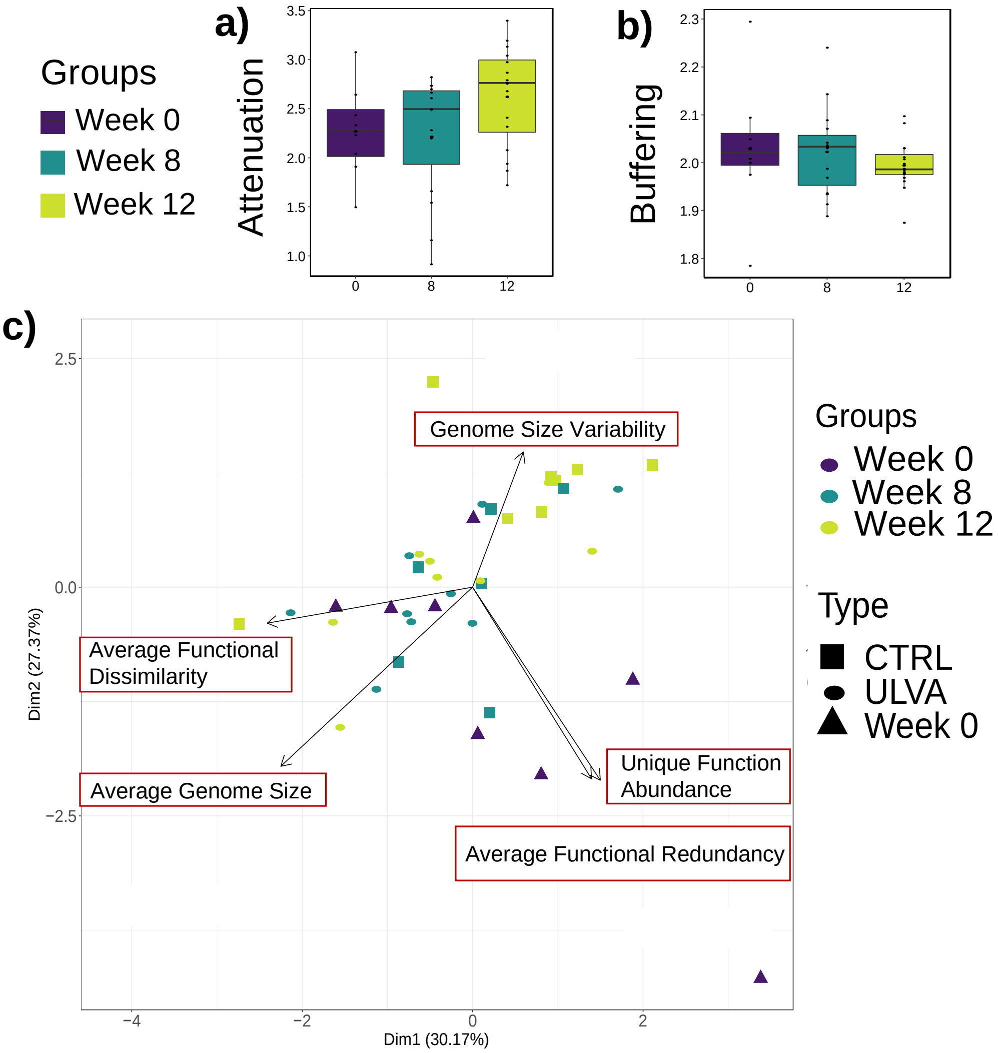

Supplementary Figure 2. How robust is the gut microbiota of Atlantic cod to taxonomic perturbation?

a) Potential robustness to perturbation magnitude (attenuation) increased over time in the hindgut of juvenile Atlantic cod from Week 0 to Week 12 (with a high level of variance in the data).

b) Potential buffering capacity towards functional perturbation decreased slightly over time from Week 0 to Week 12.

c) The microbial communities in the hindgut of juvenile Atlantic cod were separated according to variation in gene distribution features (GDF) between Week 0, Week 8 and Week 12 macroalgal dietary treatments. The points represent grouped samples with 'Treatment' denoted by symbols and Time denoted by colours; Week 0 (purple), Week 8 (dark green), and Week 12 (light green). The percent variance explained by each principal component is indicated on the axis labels. The arrow directions show the direction of the GDF vectors.

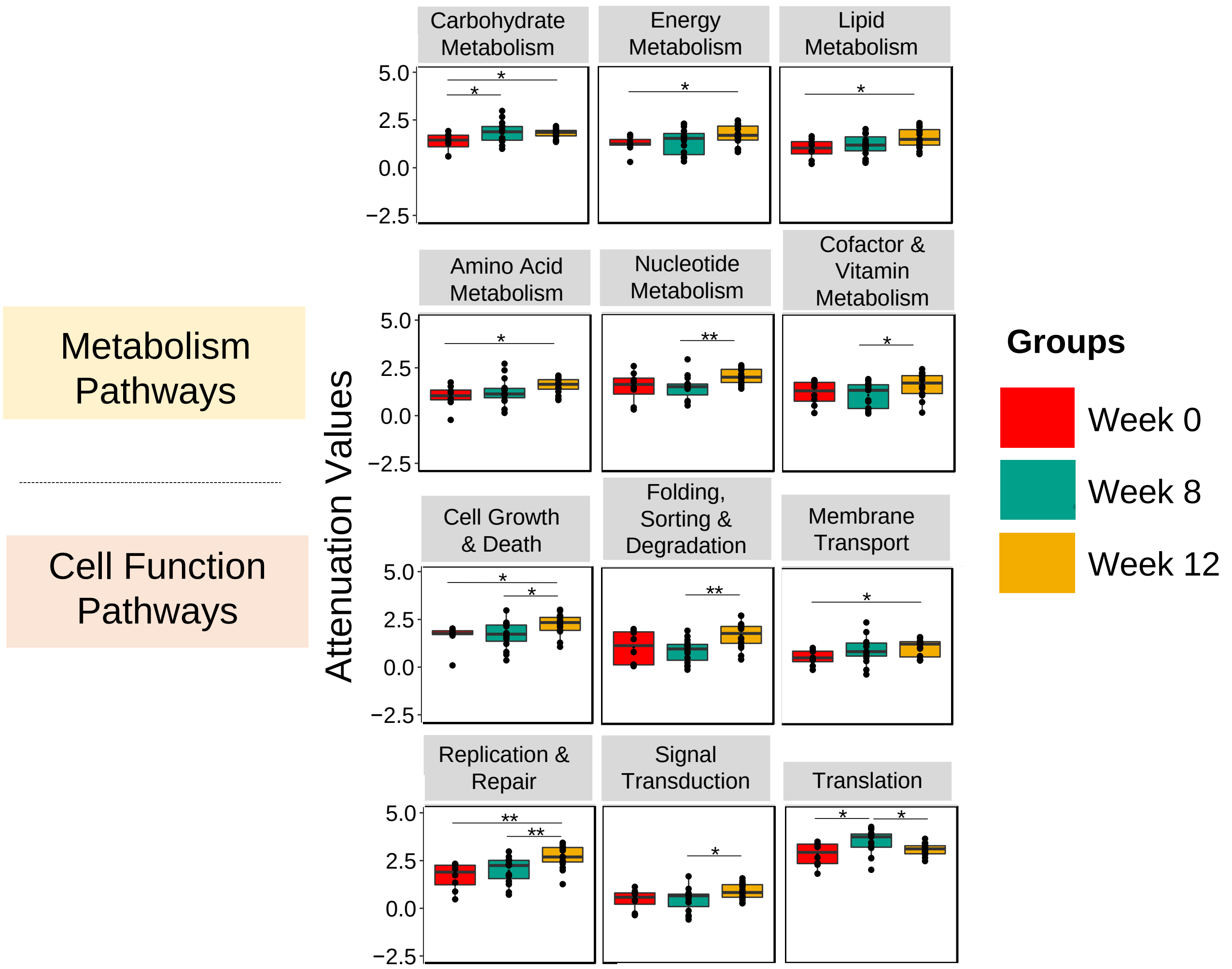

Supplementary Figure 3. How robust are specific functions in the gut microbiota of Atlantic cod over time?

Potential robustness to specific functional superpathways related to metabolism and cell function were significantly increased over time from Week 0, Week 8 and Week 12. Lines connecting categories with significant relationships (pairwise t-test) with * ($p < 0.05$), ** ($p < 0.01$), or *** ($p < 0.001$).

Supplementary Table 1. Statistical testing showing that the time-variable (Week) showed significantly different attenuation values in both an Additive model (assuming that both time and treatments are independent) and Interaction method (assuming that there is dependence between both time and treatments). These were computed using a two-way ANOVA test with * ($p < 0.05$), ** ($p < 0.01$), or *** ($p < 0.001$). Since Week was coming out to be significant for attenuation the additive model was then tested using Tukey HSD for performing multiple pair-wise comparison between the mean of the groups. For this purpose, we used a general linear hypothesis test glht() function from R's multcomp package using Tukey HSD only on Week which gives a significant difference in attenuation values between Week 8 and Week 12 $p = 0.00457^{**}$.

**Model**	**Attenuation**	**Buffering**
**Additive**	*Week* $p = 0.00398^{**}$	*Week* $p = 0.251^{NS}$
	Treatment $p = 0.14494^{NS}$	*Treatment* $p = 0.269^{NS}$
**Interaction**	*Week* $p = 0.00457^{**}$	*Week* $p = 0.246^{NS}$
	Treatment $p = 0.15486^{NS}$	*Treatment* $p = 0.262^{NS}$
	Week:Treatment $p = 0.68276^{NS}$	*Week:Treatment* $p = 0.270^{NS}$

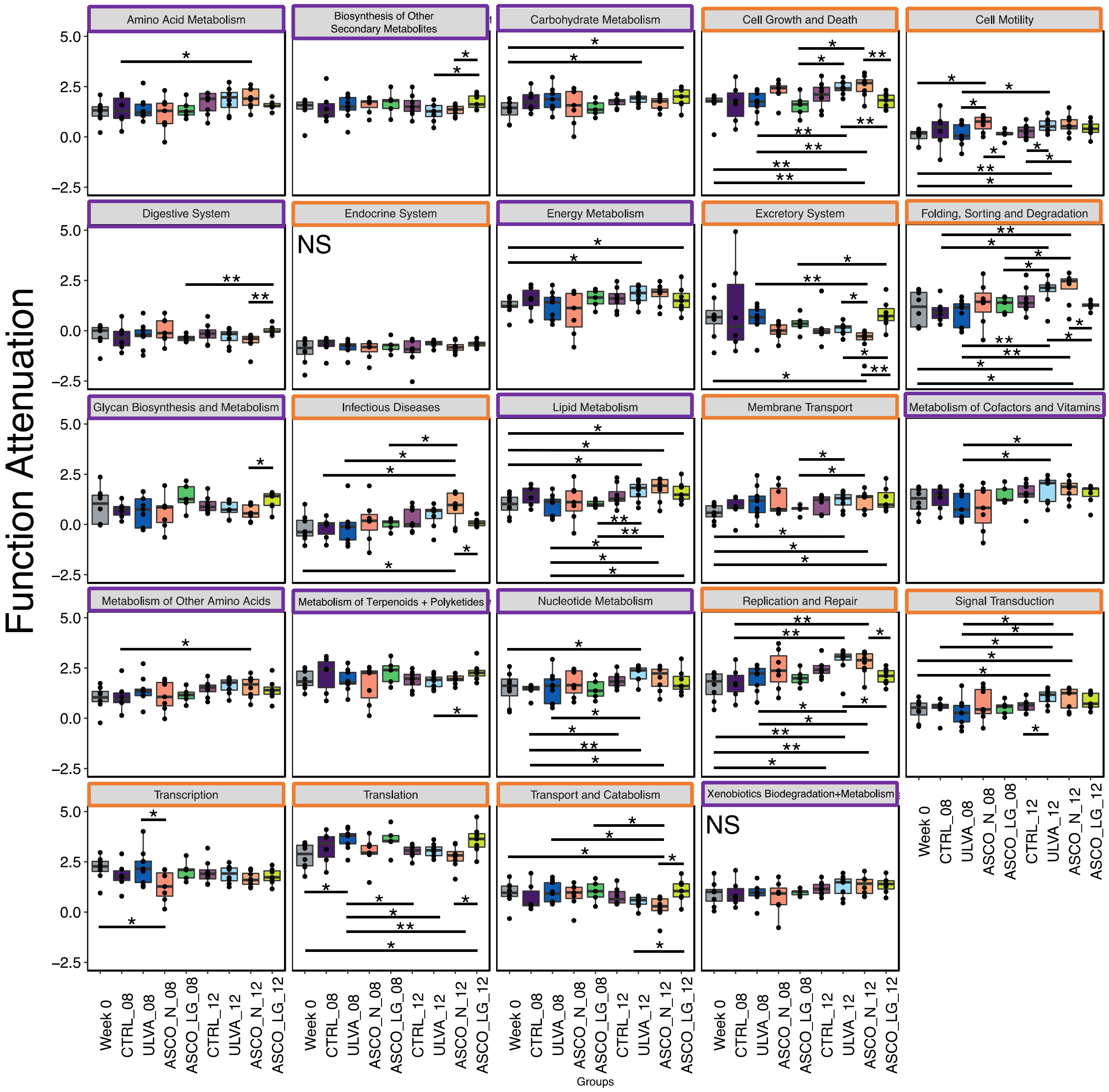

Supplementary Figure 4. How robust are specific functions in the gut microbiota of Atlantic cod across treatments?

The dietary subgroup ASCO_LG had increased values for functional superpathways related to the excretory system, translation, transport, and catabolism and reduced values for functions related to infectious diseases, cell growth and death, replication and repair. Purple boxes indicate metabolism superpathways, and orange boxes indicate cell function based superpathways. Lines connecting categories with significant relationships (pairwise t-test) with * ($p < 0.05$), ** ($p < 0.01$), or *** ($p < 0.001$).

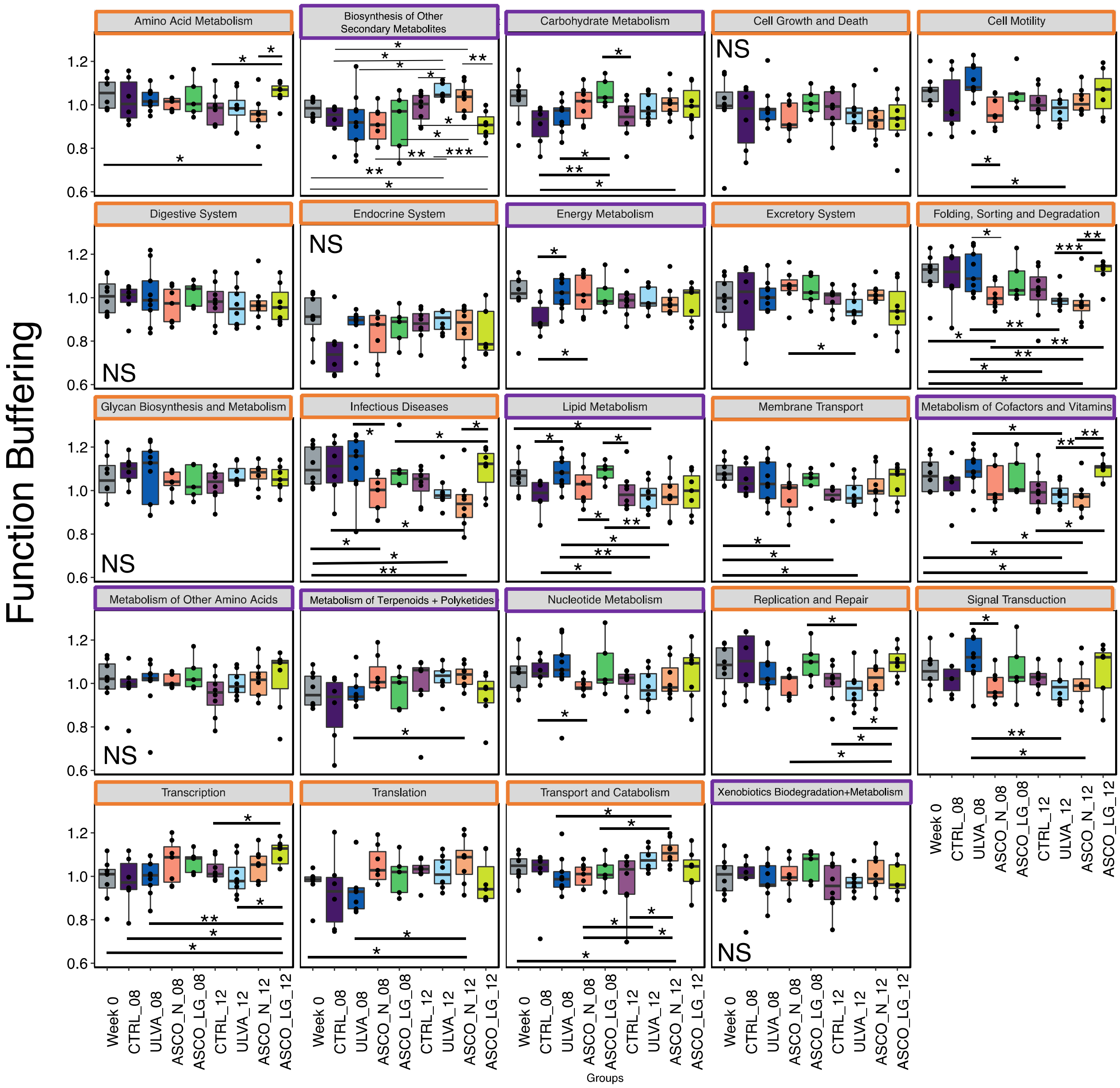

Supplementary Figure 5. What is the potential buffering capacity of specific functions in the gut microbiota of Atlantic cod across treatments?
The buffering values for superpathway functions related to metabolism (metabolism of cofactors and vitamins, biosynthesis of other secondary metabolites) decreased over time and across all treatment groups, except ASCO_LG. Purple boxes indicate metabolism superpathways, and orange boxes indicate cell function based superpathways. Lines connecting categories with significant relationships (pairwise t-test) with * ($p < 0.05$), ** ($p < 0.01$), or *** ($p < 0.001$).

Supplementary Methods.

Bioinformatics
Raw sequences were submitted to the SRA database under Bioproject Submission PRJNA636649. The subsequent paired-end reads were demultiplexed and converted to FastQ files. The sequence reads were filtered using Sickle (v1.2)[1] by applying a sliding window approach and trimming regions below a quality score of 20. Pandaseq (v 2.4)[2] was used to merge the forward and reverse reads into a single sequence. After obtaining the consensus sequences from each sample, UPARSE (v7.0.1001)[3] was used for operational taxonomic unit (OTU) construction. Reads from different samples were pooled together and barcodes were added to keep an account of sample origin. Reads were then de-replicated and sorted by decreasing abundance and singletons were discarded. Reads were then clustered based on 97% similarity, which was

followed by de novo chimera removal from most abundant sequences. Additionally, a reference-based chimera filtering step was employed to remove chimeras that may have been missed in the previous step[4]. We obtained a total of 3612 operational taxonomic units (OTUs). The assign_taxonomy.py script from the Qiime workflow was used to taxonomically classify the representative OTUs against the SILVA SSU Ref NR database release (v123)[5,6]. These taxonomic assignments were then integrated with the abundance table using the make_otu_table.py function from the Qiime workflow to produce a biom file. To find the phylogenetic distances between OTUs, the OTUs were multisequence aligned against each other using MAFFT[7]. FastTree (v2.1.7) was then used to generate an approximate maximum-likelihood phylogenetic tree in NEWICK format[8].

Supplementary References.

1. Joshi NA, Fass JN. (2011). Sickle: A sliding-window, adaptive, quality-based trimming tool for FastQ files. (Version 1.33) [Software]. Available at https://github.com/najoshi/sickle.

2. Masella AP, Bartram AK, Truszkowski JM, Brown DG, Neufeld JD. PANDAseq: paired-end assembler for illumina sequences. BMC Bioinformatics. 2012;13(1):31.

3. Edgar RC. UPARSE: highly accurate OTU sequences from microbial amplicon reads. Nat Methods. 2013;10(10):996–8.

4. Edgar RC, Haas BJ, Clemente JC, Quince C, Knight R. UCHIME improves sensitivity and speed of chimera detection. Bioinformatics. 2011;27(16):2194–200.

5. Caporaso JG, Kuczynski J, Stombaugh J, Bittinger K, Bushman FD, Costello EK, et al. QIIME allows analysis of high-throughput community sequencing data. Nat Methods. 2010;7(5):335–6.

6. Quast C, Pruesse E, Yilmaz P, Gerken J, Schweer T, Yarza P, et al. The SILVA ribosomal RNA gene database project: improved data processing and web-based tools. Nucleic Acids Research. 2012;41(D1):D590–6.

7. Katoh K, Standley DM. MAFFT Multiple Sequence Alignment Software Version 7: Improvements in Performance and Usability. Mol Biol Evol. 2013 Apr 1;30(4):772–80.

8. Price MN, Dehal PS, Arkin AP. FastTree 2–approximately maximum-likelihood trees for large alignments. PloS one. 2010;5(3).
