# Supplementary material for: Drivers of ecological assembly in the hindgut of Atlantic Cod fed a macroalgal supplemented diet
Source: NPJ Biofilms Microbiomes. 2022 May 4;8:36. doi: 10.1038/s41522-022-00296-x (PMC9068720; doi:10.1038/s41522-022-00296-x)
Supplement: Supplementary file 2 — Reporting Summary [file 41522_2022_296_MOESM2_ESM.pdf]

nature portfolio

Corresponding author(s): Umer Zeeshan Ijaz

Last updated by author(s): Dec 22, 2021

Reporting Summary

Nature Portfolio wishes to improve the reproducibility of the work that we publish. This form provides structure for consistency and transparency in reporting. For further information on Nature Portfolio policies, see our Editorial Policies and the Editorial Policy Checklist.

Statistics

For all statistical analyses, confirm that the following items are present in the figure legend, table legend, main text, or Methods section.

n/a	Confirmed	
☐	☒	The exact sample size (*n*) for each experimental group/condition, given as a discrete number and unit of measurement
☐	☒	A statement on whether measurements were taken from distinct samples or whether the same sample was measured repeatedly
☐	☒	The statistical test(s) used AND whether they are one- or two-sided<br>*Only common tests should be described solely by name; describe more complex techniques in the Methods section.*
☐	☒	A description of all covariates tested
☐	☒	A description of any assumptions or corrections, such as tests of normality and adjustment for multiple comparisons
☐	☒	A full description of the statistical parameters including central tendency (e.g. means) or other basic estimates (e.g. regression coefficient) AND variation (e.g. standard deviation) or associated estimates of uncertainty (e.g. confidence intervals)
☐	☒	For null hypothesis testing, the test statistic (e.g. *F*, *t*, *r*) with confidence intervals, effect sizes, degrees of freedom and *P* value noted<br>*Give P values as exact values whenever suitable.*
☒	☐	For Bayesian analysis, information on the choice of priors and Markov chain Monte Carlo settings
☒	☐	For hierarchical and complex designs, identification of the appropriate level for tests and full reporting of outcomes
☒	☐	Estimates of effect sizes (e.g. Cohen's *d*, Pearson's *r*), indicating how they were calculated

Our web collection on statistics for biologists contains articles on many of the points above.

Software and code

Policy information about availability of computer code

Data collection	We have used all freely and opensource code available. The raw sequences are also available in the SRA database under Bioproject Submission PRJNA636649
Data analysis	Data analysis is done in consistence with open source software particulary, those that are available at CRAN, and some that are originating from Dr Ijaz's lab, particularly, microbiomeSeq https://github.com/umerijaz/microbiomeSeq

For manuscripts utilizing custom algorithms or software that are central to the research but not yet described in published literature, software must be made available to editors and reviewers. We strongly encourage code deposition in a community repository (e.g. GitHub). See the Nature Portfolio guidelines for submitting code & software for further information.

Data

Policy information about availability of data

All manuscripts must include a data availability statement. This statement should provide the following information, where applicable:

- Accession codes, unique identifiers, or web links for publicly available datasets
- A description of any restrictions on data availability
- For clinical datasets or third party data, please ensure that the statement adheres to our policy

The raw sequences are also available in the SRA database under Bioproject Submission PRJNA636649

Field-specific reporting

Please select the one below that is the best fit for your research. If you are not sure, read the appropriate sections before making your selection.

☐ Life sciences ☐ Behavioural & social sciences ☒ Ecological, evolutionary & environmental sciences

For a reference copy of the document with all sections, see nature.com/documents/nr-reporting-summary-flat.pdf

Ecological, evolutionary & environmental sciences study design

All studies must disclose on these points even when the disclosure is negative.

Study description	Key Drivers of Ecological Assembly in the Hindgut of Atlantic Cod (Gadus morhua) when Fed with a Macroalgal Supplemented diet – How Robust Is the Gut to Taxonomic Perturbation?
Research sample	Internal and external cues shape the microbiota in the gastrointestinal tract of any species in addition to random events, which can be difficult to disentangle from a range of interacting variables. Typically, ecological approaches applied to microbial community assembly are used to elucidate these drivers. In our study, farmed Atlantic cod (Gadus morhua) were fed a diet of 10% macroalgae supplement (Ulva rigida species [ULVA] or Ascophyllum nodosum [ASCO] or a non-supplemented control diet [CTRL]) over 12 weeks. We then determined the influence of ecological assembly processes using a suite of null-modelling tools. We observed dissimilarity in the abundance of common OTUs over time, which was driven by deterministic assembly processes, In contrast, the presence or absence of OTUs was driven by stochastic assembly. The CTRL samples showed selection (variable selection - divergence in communities related to selective environmental conditions) as a critical assembly process, while dispersal limitation (limited movement results in divergent communities through stochastic processes) was a driver of the gut microbiome for fish fed the macroalgae supplemented diet at Week 12 (i.e., ASCO and ULVA). Fish from the ASCO grouping diverged into ASCO_N (normal) and ASCO_LG (lower growth), where ASCO_LG individuals found the diet unpalatable. The recruitment of new taxa over time was altered in the ASCO_LG fish, with the gut microbiome showing phylogenetic under dispersion (nepotistic recruitment of species). Finally, the gut microbiome (CTRL and ULVA only) showed increasing robustness to taxonomic disturbance over time and lower functional redundancy. This study advances our understanding of the ecological assembly and succession in the hindgut of juvenile Atlantic cod across dietary treatments. Understanding the processes driving ecological assembly in the gut microbiome, in fish research specifically, could allow us to manipulate the microbiome for improved health or resilience to disease for improved aquaculture welfare and production.
Sampling strategy	A full description of the experimental design, fish feed formulation, fish condition, sample collection and sequencing analysis is included in our previous work21. Briefly, juvenile Atlantic cod (G. morhua) were hand-graded (123 ± 7 g, SD) and randomly allocated to one of nine experimental tanks (three tanks per treatment and 60 fish per tank). Fish were acclimated for one week on a commercial fishmeal diet (Amber Neptun, Skretting, Stavanger, Norway), noted as the Week 0 sampling phase. Then tanks were assigned to the following diets, a 10% dietary macroalgae supplement (either Ulva rigida [ULVA] or Ascophyllum nodosum [ASCO] species) or a control diet for basal comparison, i.e., no algal addition [CTRL]. At Week 8 within the feed trial, a subgroup of the ASCO fish displaying ‘reduced growth rates were observed and was likely due to reduced acceptance of the feed21. We therefore subcategorised this group as [ASCO_LG], and the remaining fish with ‘normal’ growth were referred to as [ASCO_N] (see Keating et al 202121 for further discussion). The feed trial was carried out for twelve weeks.
Data collection	16S rRNA dataset collected and uploaded to public repository
Timing and spatial scale	The timepoints from week 0 to week 8 and week 12
Data exclusions	NA
Reproducibility	NA
Randomization	NA
Blinding	NA

Did the study involve field work? ☒ Yes ☐ No

Field work, collection and transport

Field conditions	See manucript
Location	See manuscript
Access & import/export	NA
Disturbance	*Describe any disturbance caused by the study and how it was minimized.*

Reporting for specific materials, systems and methods

We require information from authors about some types of materials, experimental systems and methods used in many studies. Here, indicate whether each material, system or method listed is relevant to your study. If you are not sure if a list item applies to your research, read the appropriate section before selecting a response.

Materials & experimental systems

n/a	Involved in the study
☒	☐ Antibodies
☒	☐ Eukaryotic cell lines
☒	☐ Palaeontology and archaeology
☐	☒ Animals and other organisms
☒	☐ Human research participants
☒	☐ Clinical data
☒	☐ Dual use research of concern

Methods

n/a	Involved in the study
☒	☐ ChIP-seq
☒	☐ Flow cytometry
☒	☐ MRI-based neuroimaging

Animals and other organisms

Policy information about studies involving animals; ARRIVE guidelines recommended for reporting animal research

Laboratory animals	Atlantic Cod
Wild animals	They were rared from wild brood stocks.
Field-collected samples	Please refer to https://animalmicrobiome.biomedcentral.com/articles/10.1186/s42523-020-00065-1
Ethics oversight	Please refer to https://animalmicrobiome.biomedcentral.com/articles/10.1186/s42523-020-00065-1

Note that full information on the approval of the study protocol must also be provided in the manuscript.
